# How emotion is experienced and expressed in multiple cultures: a large-scale experiment across North America, Europe, and Japan

**DOI:** 10.3389/fpsyg.2024.1350631

**Published:** 2024-06-20

**Authors:** Alan S. Cowen, Jeffrey A. Brooks, Gautam Prasad, Misato Tanaka, Yukiyasu Kamitani, Vladimir Kirilyuk, Krishna Somandepalli, Brendan Jou, Florian Schroff, Hartwig Adam, Disa Sauter, Xia Fang, Kunalan Manokara, Panagiotis Tzirakis, Moses Oh, Dacher Keltner

**Affiliations:** ^1^Hume AI, New York, NY, United States; ^2^Department of Psychology, University of California, Berkeley, Berkeley, CA, United States; ^3^Google Research, Mountain View, CA, United States; ^4^Advanced Telecommunications Research Institute, Kyoto, Japan; ^5^Graduate School of Informatics, Kyoto University, Kyoto, Japan; ^6^Department of Electrical Engineering, University of Southern California, Los Angeles, CA, United States; ^7^Faculty of Social and Behavioural Sciences, University of Amsterdam, Amsterdam, Netherlands; ^8^Zhejiang University, Zhejiang, China

**Keywords:** facial expressions, emotion, emotional expression, emotional expression and experience, machine learning

## Abstract

Core to understanding emotion are subjective experiences and their expression in facial behavior. Past studies have largely focused on six emotions and prototypical facial poses, reflecting limitations in scale and narrow assumptions about the variety of emotions and their patterns of expression. We examine 45,231 facial reactions to 2,185 evocative videos, largely in North America, Europe, and Japan, collecting participants’ self-reported experiences in English or Japanese and manual and automated annotations of facial movement. Guided by Semantic Space Theory, we uncover 21 dimensions of emotion in the self-reported experiences of participants in Japan, the United States, and Western Europe, and considerable cross-cultural similarities in experience. Facial expressions predict at least 12 dimensions of experience, despite massive individual differences in experience. We find considerable cross-cultural convergence in the facial actions involved in the expression of emotion, and culture-specific display tendencies—many facial movements differ in intensity in Japan compared to the U.S./Canada and Europe but represent similar experiences. These results quantitatively detail that people in dramatically different cultures experience and express emotion in a high-dimensional, categorical, and similar but complex fashion.

## Introduction

Suppose a film routinely arouses laughs, cries, or grimaces. What can we infer about how it makes people feel? Answers to this question remain scientifically elusive (Barrett et al., 2019; Cowen A. et al., 2019; Keltner et al., 2019). For the past 50 years, tracing back to a classic study by Ekman and Friesen (1971), scientific efforts have largely sought to illuminate the nature of emotional expression in the search for one-to-one mappings between six “basic” emotions—anger, disgust, fear, happiness, sadness, and surprise—and prototypical facial expressions (Elfenbein and Ambady, 2002; Lench et al., 2011; Durán et al., 2017; Barrett et al., 2019). While other lines of work have attempted to map facial expressions to appraisal dimensions (Scherer, 2013) or to a small number of other categories, debates about the relationship between emotions and facial expressions have largely been oriented around prototypical facial expressions associated with these six states.

However, emotional expression is far more complex. Recent studies have established that the varieties of emotional experience and expression are upwards of four times more complex than represented in studies of six emotions (Cowen and Keltner, 2017, 2019, 2020; Shiota et al., 2017; Cowen et al., 2018, 2020; Saarimäki et al., 2018; Cowen A. S. et al., 2019; Cowen A. et al., 2019; Horikawa et al., 2020). People reliably report feeling dozens of other emotions such as “awe,” “excitement,” and “relief” in a wide range of circumstances (Cowen and Keltner, 2017; Cowen et al., 2020) and perceive most of these emotions in expressive behavior in the face and voice (Shiota et al., 2017; Cordaro et al., 2018, 2020; Cowen and Keltner, 2020). The six “basic” emotions capture under a third of the variance in people’s diverse, systematic responses to emotion antecedents and expressions (Cowen A. et al., 2019). Methodological advances now enable computational approaches to the measurement of facial expression (Dupré et al., 2020; Cowen et al., 2021) and data-driven approaches have enabled a more fine-grained quantitative understanding of variability in facial expressions across individuals, situations, and cultures (Jack et al., 2012, 2016; Cowen et al., 2021; Le Mau et al., 2021). Taken together, these methodological and empirical advances reveal that studies seeking to map prototypical expressions onto experiences of the six “basic” emotions only capture a fraction, at best, of what facial expressions reveal. There is much to learn about how people move their faces when feeling specific emotions.

**Figure 1:**
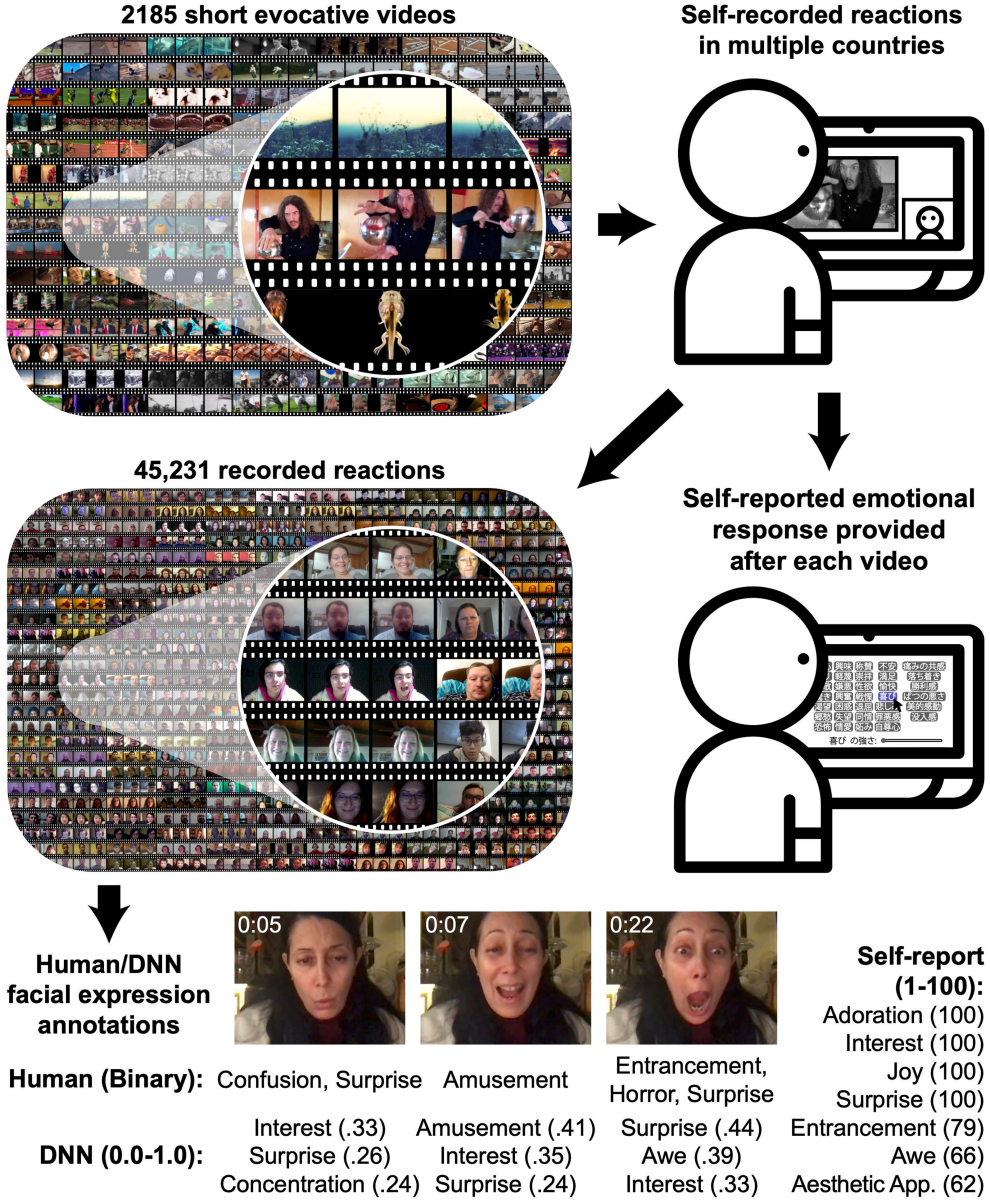
The Berkeley Reactions to Affective Video Elicitors (BRAVE) database. 2,185 short evocative videos (Scherer, 2013) were shown to participants in multiple countries, who recorded their reactions and reported on their emotional experiences. Each of 45,231 resulting reaction videos was annotated by human raters (2 ratings per 2 s segment) in terms of 42 perceived emotions and by a deep neural network in terms of 30 predicted human judgments (6 annotations per second).

Understanding how people express emotion requires studying a much wider range of behaviors with inductive, data-driven analytic approaches. Here we introduce the Berkeley Reactions to Affective Video Elicitors (BRAVE) database (Figure 1), a corpus of 45,231 recordings of participants largely from North America, Europe, and Japan reacting to 2,185 validated, evocative short videos and reporting on their emotional experiences. BRAVE is orders of magnitude larger than previously published databases of recorded reactions to emotional stimuli. The videos presented to participants are diverse, naturalistic, and emotionally varying and evocative, including scenes from across life such as vast landscapes, births, funerals, accidents, historic footage, practical jokes, endearing images of babies and pets, nature scenes, and artwork (see Methods). Moreover, the reactions feature individuals from cultural groups with disparate histories, languages, belief systems, norms, and values (Scherer et al., 1988; Kitayama et al., 2000; Rothbaum et al., 2000; Lim, 2016). By analyzing thousands of naturalistic reactions to rich, diverse antecedents, we paint a detailed portrait of how people experience and express emotion.

What emotions do people in different cultures experience when viewing evocative videos? What facial movements do they produce? How are feelings and expressions related? Existing datasets and techniques – limited by small sample sizes and a lack of generalizability – have not yet enabled a direct investigation into these questions. To answer these questions, we applied a recently developed framework for investigating representational spaces of emotion-related behavior (Cowen and Keltner, 2018; Cowen A. et al., 2019; Keltner et al., 2019), automated methods of quantifying facial expressions (Cowen et al., 2021), and data-driven statistical modeling approaches to the BRAVE dataset. This framework – called Semantic Space Theory (SST) – has recently proven useful in mapping the multidimensional space of emotions distinguishable in self-report, expressive behavior in the face and voice, visual and musical artistic depictions, and more (Cowen and Keltner, 2021; Keltner et al., 2022). SST leverages recent advances in machine learning and statistical modeling to characterize data-driven taxonomies of emotion. Emotion-related responses are characterized in terms of dynamic trajectories within a semantic space that captures how emotions, eliciting stimuli, and expressive behaviors relate to one another. Given a large amount of data in a given modality, we can use inductive statistical modeling to characterize the *dimensionality* of the space – the number of distinct kinds of emotion organizing the semantic space – successfully applied to characterizing the semantic spaces of facial expressions in photographs (Cowen and Keltner, 2019), artwork (Cowen and Keltner, 2020), in different social contexts (Cowen et al., 2021), and in response to music (Cowen et al., 2020). In multiple cases this approach has been used to measure similarities and differences in the semantic spaces of emotion across different cultures (Cowen et al., 2020), which can be compared directly if they measure the same emotion-related features (see Results and Methods for more detail). But the SST framework has not yet been applied to understanding the relationship between dynamic facial actions and self-reported emotional experience, a central issue in the study of emotional expression (Durán and Fernández-Dols, 2021; Witkower et al., 2023). The BRAVE dataset presents a unique opportunity to apply the SST framework to directly measure the semantic space of facial expressions and self-reported emotional experience at extremely large scale, across different cultures, and building upon recent methodological and theoretical developments.

This study allowed us to directly address questions of long-standing interest to the field: what is the relationship between facial expressions and underlying emotional states, and how does this relationship vary between cultures? Our findings reveal how (a) a rich, high-dimensional space of emotional experience and expression is shared across different cultural groups, (b) a wide range of self-reported emotions can accurately be inferred from facial expressions at the aggregate – but more modestly so at the individual – level, (c), members of different cultures converge to a high degree in the facial muscle patterns associated with 12 emotions, and (d) culture-specific display tendencies explain dramatic differences in the intensity of facial movements. These results greatly advance our understanding of how people in different cultures use their faces to express emotion.

## Materials and methods

### Participants

Participants of the English-language survey were recruited via Amazon Mechanical Turk and Prolific. Japanese participants were recruited via Crowdworks. Participants were eligible for participation if they had a working webcam as well as linguistic proficiency in the sampled languages (English or Japanese). On average, a total of 18.7 English-language and 7.7 Japanese survey participants provided ratings and/or reactions for each of the 2,185 videos. English-language survey participants (*n* = 1,332, 678 male [124 unreported], mean age 31.8) responded to 30.7 videos on average and Japanese survey participants (*n* = 61, 39 male [6 unreported], mean age 38.4) responded to 274.3 videos on average. Given that the average Japanese participant responded to a greater number of videos, it is important to note that the intensity of their expressions did not decline over time (Supplementary Figure 6). English-language survey participants originated from the U.S. (*n* = 568), U.K. (*n* = 158), Portugal (*n* = 96), Poland (*n* = 94), Canada (*n* = 55), Mexico (*n* = 35), Italy (*n* = 29), Greece (*n* = 28), Spain (*n* = 26), France (*n* = 11), Nigeria (*n* = 10), Australia (*n* = 8), New Zealand (*n* = 8), India (*n* = 6), Israel (*n* = 3), other Europe (*n* = 62), other Latin America (*n* = 16), and other Africa (*n* = 6) (113 unreported; 9 from East or Southeast Asia were excluded). The U.S./Canada/Europe sample used in certain analyses comprises 92% (*n* = 1,127) of English-language survey participants who reported their country of origin. All Japanese survey participants originated from Japan (6 unreported).

### Videos

The 2,185 video stimuli were drawn from Cowen and Keltner (2017). The videos were originally gathered by querying search engines and content aggregation websites for phrases and situations related to the 34 emotion categories measured in the study.

### Surveys

In a given survey, participants viewed 15–30 randomly selected videos and used a webcam plugin to record their reactions. Each video was repeated so that its final duration was at least 8 s. Recording began at the start of each video and was stopped 1 s after the end of each video. During the experimental trials participants were presented with the following instructions in a small box at the top of their screen: “When watching the videos, make sure your entire face is visible | If your webcam stops working, click the ‘Reset’ button and then click the box to reset recording. | Use your face expressively. React in a way that would allow others to understand your feelings. | Choose any many buttons as needed to describe your emotional response.” The instructions to react expressively were included after some participants provided feedback to pilot versions of the study indicating that they had suppressed their expressive reactions.

Participants in the emotion category ratings surveys selected from 34 emotion categories to describe their response to each video. The 34 emotion categories were drawn from Cowen and Keltner (2017) (for a full list of emotion terms, their translations into Japanese, and related references, see Table S1 in Supplementary material). For each emotion category selected, participants reported the intensity of the emotion on a 1–100 scale. Participants in the valence and arousal ratings surveys rated the valence and arousal evoked by each video on bipolar 1–9 Likert scales.

**Figure 2:**
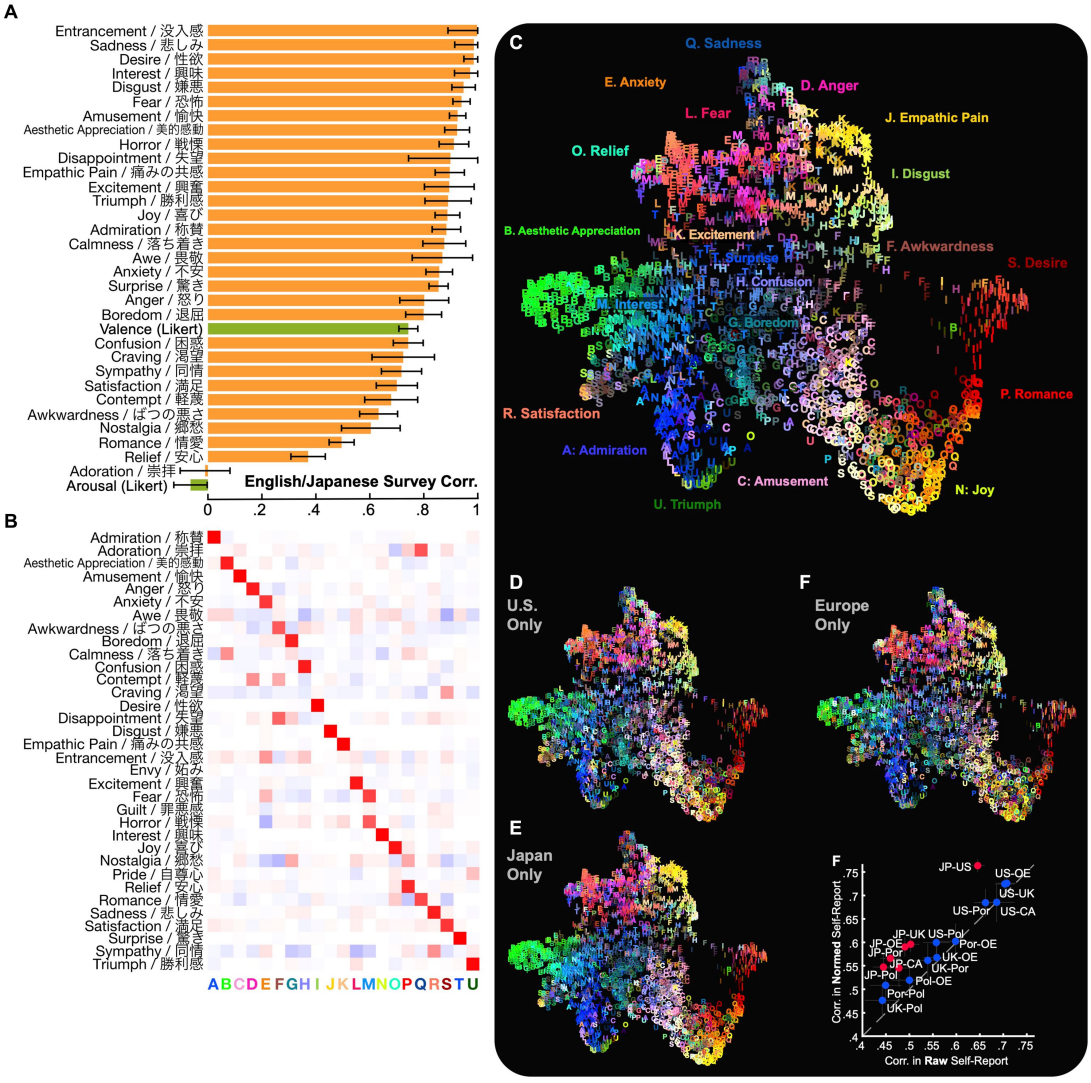
The experience of emotion in multiple cultures and languages. **(A)** Specific feelings evoked by video were well preserved across English-language and Japanese self-report. To examine whether reported emotional experiences were preserved across multiple cultures and languages, we computed correlations between ratings in English-language and Japanese responses and adjusted for within-language variation (see Methods). Reports of many specific categories of emotion (e.g., “interest”) were better preserved than ratings of valence and arousal. **(B)** 21 distinct dimensions of emotional experience were preserved across surveys. To examine how many distinct self-reported emotions were preserved, we applied principal preserved components analysis (PPCA) between ratings across languages. This analysis revealed 21 significant dimensions (*q* < 0.005 across all held-out raters, *q* < 0.05 across held-out raters in each culture individually), ForwardStop sequential FDR-corrected (Rothbaum et al., 2000) one-tailed Wilcoxon signed-rank test (Cowen and Keltner, 2018). The dimensions were subjected to factor rotation to characterize the different kinds of self-reported emotional experience evoked by videos, captured in both English-language and Japanese responses. **(C–E)** The distribution of emotional experience across videos is similar in the U.S. and Japanese responses. To visualize the distribution of emotional experiences, we applied uniform manifold approximation and projection [UMAP (McInnes, 2018)] to the concatenated mean ratings in each language. Each video is assigned a letter corresponding to its maximal dimension [from **(B)**] and a color based on its average rating across all responses **(C)** or within the U.S. or Japan only **(D/E)**. Emotional experiences were remarkably similar across different languages and countries, despite lexical and cultural differences. (See also the interactive map: https://tinyurl.com/yywa7kjf). **(F)** Country-wise similarity of self-report judgments. Responses in Poland and Portugal are slightly less similar to other countries (though as we will see, findings are consistent for facial expression [Figure 5C]). Normalization of self-report values (subtracting mean and dividing by standard deviation) within each country slightly increases correlations between English-language and Japanese responses, a pattern we will see reflected more strongly in nonverbal expression (Figure 5C). CA, Canada; JP, Japan; OE, Other Europe; Pol, Poland; Por, Portugal; UK, United Kingdom; US, United States.

Participants on Amazon Mechanical Turk and Crowdworks could perform as many surveys as desired with different videos in each. Participants on Prolific were limited to one survey. The experimental procedures were approved by the Institutional Review Board at the University of California, Berkeley and all research was performed in accordance with relevant guidelines and regulations. All participants gave their informed consent to participate in experiment and consent to publish their identifiable images. See Supporting Information for further details regarding the surveys. Surveys were translated into Japanese by a team of bilingual researchers (see Acknowledgements), with particular care provided in translating emotion terms and questions about valence and arousal (see Figure 2 for correlations between English-language and Japanese surveys, Table S1 in Supplementary material for references).

### Correlations between cultural groups

We computed the correlations between ratings of each category across cultural groups (countries or survey languages). To do so, for each rating (emotion category or valence/arousal dimension), we first drew individual ratings at random from each cultural group. One rating or score was drawn for each of the 2,185 videos. For instance, when comparing ratings of “disgust,” one rating of each video was drawn from each cultural group (each ranging from 0 to 100). We then computed the correlation in the ratings between the cultural group, across the 2,185 videos. Finally, we divided by the maximum attainable correlation, or noise ceiling, based on within-culture variability of the ratings. As explained further below, the maximum attainable correlation is estimated by computing the geometric mean of the correlations between individual ratings drawn at random from within the same cultural group (Figure 1).

### Principal preserved component analysis

PPCA extracts shared dimensions that maximize the covariance between two parallel datasets (e.g., emotions ratings). To do so, PPCA first seeks a unit vector α_1_ that maximizes the objective function


$$\mathrm{Cov}\left(X\alpha_{1}\mathrm{,Y}\alpha_{1}\right)$$

in which we call α_1_ the first principal preserved component. Subsequent components are obtained by seeking additional unit vectors α_i_ that maximize the objective function Cov (Xα_i_, Yα_i_) subject to the constraint that α_1_ is orthogonal to the previous components, α_1_,…, α_i-1_.

In the special case that X = Y, PPCA is equivalent to PCA, given that the latter method maximizes the objective function


$$\mathrm{Var}\left(X\alpha_{i}\right)\mathrm{=Cov}\left(X\alpha_{i},\;\;X\alpha_{i}\right)$$

(substituting another *X* for *Y* in Cov[Xα_1_, Yα_1_]). See Video S2 in Supplementary material for illustration of why the PCA objective is ill-suited to our aims. Also note the similarity to the PLSC objective, which seeks to find two separate bases and ß to maximize Cov(Xα_i_, Yß_i_), as well as the CCA objective, which seeks to maximize Corr(Xα_i_, Yß_i_). However, given our aim of finding *preserved* dimensions across ratings (between cultures, or predicted vs. reported), PPCA derives only one basis, α, that applies to both datasets. (In PPCA, therefore, the data matrices must be commensurate: observations in both datasets must be of the same dimension; i.e. the number of rows in X and Y must be equal).

To solve the PPCA objective and find an α_1_ we apply eigendecomposition to the addition of the cross-covariance matrix between datasets and its transpose: Cov(X,Y)/2 + Cov(Y,X)/2. We claim that the principal eigenvector of this symmetric matrix maximizes Cov(Xα_1_, Yα_1_). To derive this, first recall a general property of cross-covariance, Cov(Xa, Yb) = b^T^Cov(X, Y)a. Thus,


$$\mathrm{Cov}\left(X\alpha_{1}\mathrm{,Y}\alpha_{1}\right)\;\;=\;\;\alpha_{1}{\;}^{T}\mathrm{Cov}\left(\mathrm{X,Y}\right)\;\alpha_{1}$$

(Property 1)

In addition, because both Xα_1_ and Yα_1_ are vectors, Cov(Xα_1_, Yα_1_) = Cov(Yα_1_, Xα_1_). Thus,


$$\begin{array}{l} \mathrm{Cov}\left(X\alpha_{1},\;Y\alpha_{1}\right)\;\;\\ =\;\;\mathrm{Cov}\left(X\alpha_{1},\;Y\alpha_{1}\right)\mathrm{/2+Cov(Y}\alpha_{1},\;X\alpha_{1}\mathrm{/2} \end{array}$$

(Property 2)

Combining these two properties, we can see that.


$$\begin{array}{l} \mathrm{Cov}\left(X\alpha_{1}\mathrm{,Y}\alpha_{1}\right)\;\;\\ =\;\;\mathrm{Cov}\left(X\alpha_{1}\mathrm{,Y}\alpha_{1}\right)\mathrm{/2+Cov(Y}\alpha_{1}\mathrm{,X}\alpha_{1}\mathrm{/2} \end{array}$$

(By property 2)


$$=\;\;\alpha_{1}{\;}^{T}\mathrm{Cov}\left(\mathrm{X,Y}\right)\;\alpha_{1}\mathrm{/2+}\alpha_{1}{\;}^{T}\mathrm{Cov}\left(\mathrm{Y,X}\right)\;\alpha_{1}\mathrm{/2}$$

(By property 1)


$$=\alpha_{1}{\;}^{T}\left[\mathrm{Cov}\left(\mathrm{X,Y}\right)\mathrm{/2+Cov}\left(\mathrm{Y,X}\right)\mathrm{/2}\right]\;\alpha_{1}$$

Now, letting R = [Cov(X,Y)/2 + Cov(Y,X)/2], we see that maximizing α_1_^T^Rα_1_ is equivalent to maximizing Cov(Xα_1_, Yα_1_), the originally stated PPCA objective. (Note that if X = Y, we are applying eigendecomposition to Var[Xα_i_] = Cov[Xα_i_, Xα_i_], which performs PCA.)

Finally, the min-max theorem dictates that the principal eigenvector of R maximizes α_1_^T^Rα_1_ subject to α_1_ being a unit vector (||α_1_|| = 1).

We have thus found a unit vector α_1_ that maximizes Cov(Xα_1_, Yα_1_)—the covariance between the projections of X and Y projected onto the first component. Based on the min-max theorem, subsequent eigenvectors α_i_ will maximize Cov(Xα_i_, Yα_i_) subject to their orthogonality with previous components α_1_ through α_i-1_ and to each α_i_ also being a unit vector (|α_i_| = 1).

We note that the min-max theorem also provides that the last eigenvector, α_n_, will minimize Cov(Xα_n_, Yα_n_), equivalent to maximizing-Cov(Xα_n_, Yα_n_). Hence, if there are dimensions of negative covariance between the two datasets, then some eigenvectors will maximize the negative covariance.

With respect to the corresponding eigenvalues, each eigenvalue λ_i_ will be equal to Cov(Xα_i_, Yα_i_). To see this, note that:


$$\left[\mathrm{Cov}\left(\mathrm{X,Y}\right)\mathrm{/2+Cov}\left(\mathrm{Y,X}\right)\mathrm{/2}\right]\;\alpha_{i}=\lambda_{i}\;\alpha_{i}$$

(Eigenvalue equation)


$$\alpha_{i}{\;}^{T}\;\left[\mathrm{Cov}\left(\mathrm{X,Y}\right)\mathrm{/2+Cov}\left(\mathrm{Y,X}\right)\mathrm{/2}\right]\;\alpha_{i}=\alpha_{i}{\;}^{T}\;\lambda_{i}\;\alpha_{i}$$

$$\mathrm{Cov}\left(X\alpha_{i}\mathrm{,Y}\alpha_{i}\right)\;\;=\;\lambda_{i}\;\alpha_{i}{\;}^{T}\alpha_{i}$$

(By property 1)

Now α_i_^T^α_i_ = 1 because the α_i_ are orthonormal. Hence,


$$\mathrm{Cov}\left(X\alpha_{i}\mathrm{,Y}\alpha_{i}\right)=\lambda_{i}$$

This also entails that there will be negative eigenvalues corresponding to negative covariance.

**Figure 3:**
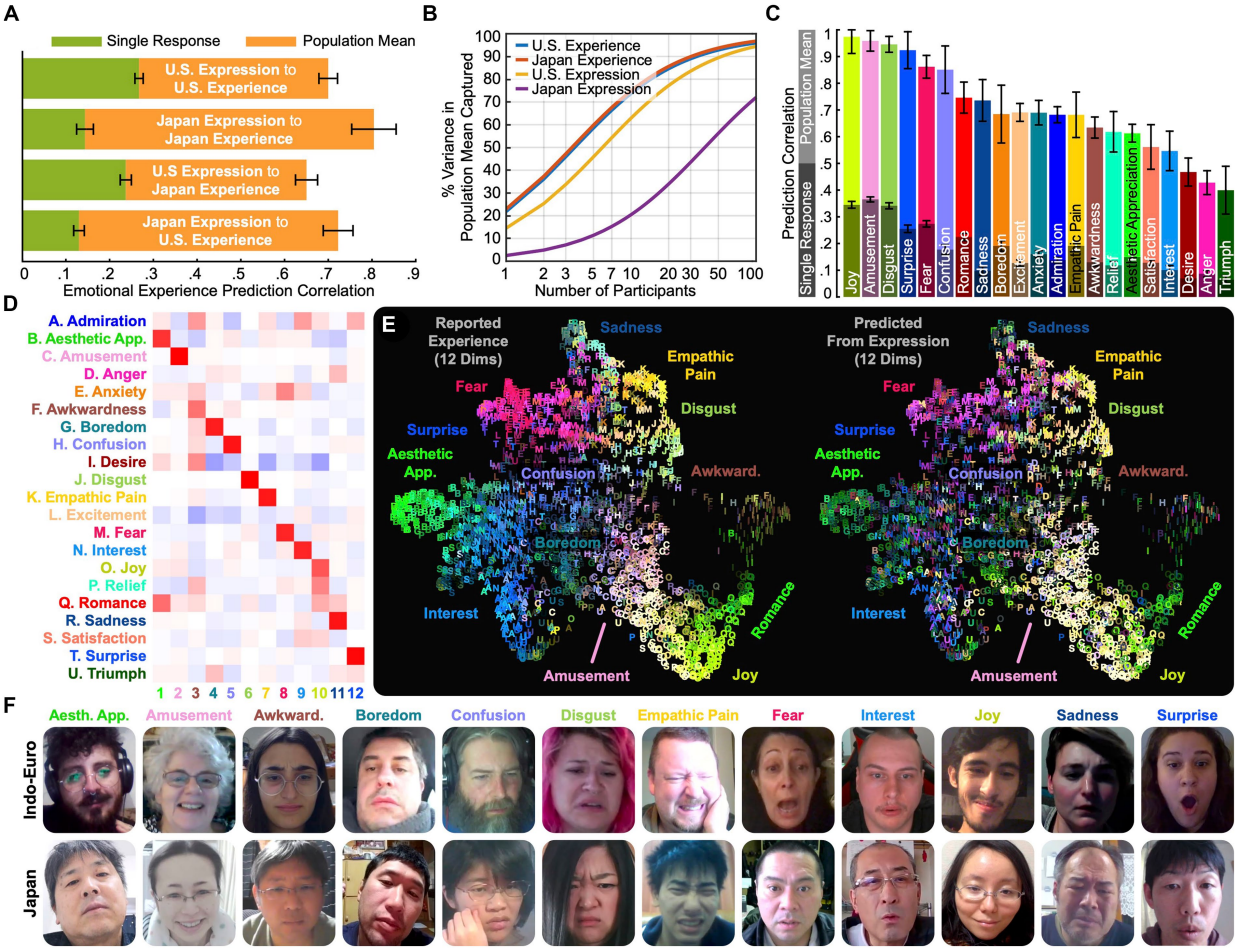
What expressions reveal. **(A)** Accuracy of reported emotional experience prediction from expression within and across cultures. Linear models were trained to predict average experience in the U.S. and Japan from expression annotations in response to the 2,185 videos. Predictions using individual expressive responses were modestly accurate (see Video S1 in Supplementary material for example predictions), but predictions using average expressions were quite accurate (*r* = 0.70 [SE 0.02] in the U.S., 0.80 [SE 0.05] in Japan). At the aggregate level, expressions are richly informative regarding emotional experience. **(B)** Degree to which average experience and expression are captured by varying sample sizes. To examine the number of responses required to predict average experience from expression, we computed the systematic variance captured by averaging across varying numbers of participants. Individual responses are highly variable, capturing 14.5% of the variance in average expression in the U.S. and 3.1% in Japan. **(C)** Prediction correlations for each of 21 dimensions of experience. Data were combined across all countries to precisely estimate correlations. **(D)** 12 distinct dimensions of emotional experience were predicted with significant accuracy. To examine how many distinct self-reported emotions were predicted accurately from expression, we applied PPCA between average predicted and reported experience. This analysis revealed 12 dimensions (*q* < 0.05 across held-out raters), ForwardStop FDR-corrected (Rothbaum et al., 2000) 1-tailed Wilcoxon signed-rank test (Cowen and Keltner, 2018). The dimensions were factor rotated (varimax) to understand which experiences corresponded to distinct expressions. Emotions loading on similar dimensions, e.g., “esthetic appreciation” and “romance,” were expressed in similar facial movements. **(E)** Distribution of 12 experience/expression dimensions across videos. Experience map (Figure 2C) colored based on reported vs. predicted experience along 12 emotion dimensions. Predicted emotional experience based on facial reactions to the majority of the 2,185 video stimuli can be explored at: https://tinyurl.com/yywa7kjf. **(F)** Example expressions yielding accurate emotion predictions. Still frames from reactions that accurately scored among the highest on each dimension (based on the product of predicted emotion and average self-report) reveal emotions were expressed in qualitatively recognizable facial movements.

We performed PPCA between the averaged self-report ratings from each culture (Figure 2) or between the averaged self-report ratings across cultures and the predicted self-report ratings from our model (Figures 3D–F). To ascertain the number of significant dimensions of covariance, we performed a leave-one-subject-out analysis, in which PPCA was iteratively performed on data from all but one participant and then the held-out ratings were projected onto the extracted dimensions and correlated. Partial Spearman correlations were used, controlling for projections onto previous dimensions, to account for possible curvilinear relationships. Wilcoxon signed-rank tests were then applied to the correlations for held out raters to test each dimension for significance. See Cowen A. S. et al. (2019) and Cowen et al. (2020). for results of repeated Monte Carlo simulations further validating these methods.

### Facial expression deep neural network

A DNN is a machine learning algorithm that learns to apply multiple layers of transformation to input data in order to predict complex outputs. We used a supervised DNN that processed the temporal trajectory of the RGB pixel values making up a video of a face over the course of up to 1 s, at a rate of 6 frames per second, to predict the proportions of raters who would attribute each label to each expression. To extract faces from video, each face was first detected within a video frame using a deep convolutional neural network, similar to the method provided by the Google Cloud Face Detection API. Faces were tracked across each video using traditional optical flow methods.

### Facial expression DNN architecture

Face-based visual features were extracted using layers from the NN2 FaceNet architecture (Russell, 2003) to characterize mid-level attributes involved in early stages of face perception. These layers consisted of an Inception (5a) block with a 7×7 feature map comprising 1,024 channels, which was fed into a 7×7 average pooling layer, generating a 1,024 dimensional vector representing face image features within a given frame of the video. The resulting features were then fed into two long short-term memory (LSTM) layers, each with 64 recurrent cells, to capture the dependence of facial expression recognition on temporally unfolding patterns of facial movement. Finally, the output of the last LSTM layer was fed into a mixture-of-experts model (two experts, plus a dummy expert). A cross entropy loss with a sigmoid activation function was used for the final layer with 30 nodes.

### Facial expression DNN training

The DNN was trained on a total of 342,546 ratings of 247,292 video clips of facial expressions independently gathered on YouTube. Clips were extracted from videos which were manually collected by raters, who were instructed to conduct a broad search for videos likely to contain emotional expressions. The facial expression clips were then rated by English speakers in India. The task was to select all facial expression categories that applied to each face.

### Facial action coding system DNN

To characterize structural aspects of the face that differentiate dimensions of emotional experience, we applied an additional supervised DNN that processed the temporal trajectory of the RGB pixel values making up a video of a face over the course of up to 1 s, at a rate of 1 frame per second, to predict the probability that a given facial movement was present in the video.

### FACS DNN architecture

We utilized layers from the FaceNet Inception Resnet v1 model (Schroff et al., 2015), pretrained on the VGGFace2 dataset (transfer learning Pan and Yang, 2010; Cao et al., 2017). We froze all layers up until the last convolutional block and unfroze the last convolutional block. On top of this architecture we added the following fresh untrained layers: 2D adaptive average pool (output_size = 1),^1^ followed by a dropout layer (*p* = 0.6). The features were then flattened and fed to a linear (1790 in features → 512 out features) layer, followed by Batchnorm1d (eps = 0.001, momentum = 0.1, affine = True) and a final linear layer (512 in features → 48 * 4 out features).

### FACS DNN training

The DNN was trained on 467,566 static images of facial expressions collected for a separate study (Brooks et al., 2024), in which web-based participants used a computer webcam to photograph themselves mimicking facial expressions. 1,500 of the original images were coded on the presence or absence of 48 Action Units (AUs) by two certified expert FACS coders.

### Manual perceptual annotations of reaction videos

We collected manual annotations on Amazon Mechanical Turk of each 2 s segment extracted from the reaction videos (the final segment for each reaction was 1–3 s). A total of 3,293 raters participated (1,599 male, mean age approx. 34.7 [based on decade ranges]). Each segment was rated by 2 raters, for a total of 649,894 ratings of 324,947 clips, in terms of 42 emotion categories [drawn from Cowen and Keltner (2019)]; “Admiration,” “Adoration,” “Esthetic Appreciation,” “Amusement,” “Anger,” “Anxiety,” “Awe,” “Boredom,” “Calmness,” “Concentration,” “Confusion,” “Contemplation,” “Contempt,” “Contentment,” “Desire,” “Disappointment,” “Disgust,” “Distress,” “Doubt,” “Ecstasy,” “Elation,” “Embarrassment,” “Empathic Pain,” “Entrancement,” “Fear,” “Horror,” “Interest,” “Love,” “Neutral,” “None,” “Nostalgia,” “Pain,” “Pride,” “Realization,” “Relief,” “Romance,” “Sadness,” “Satisfaction,” “Shame,” “Surprise,” “Sympathy,” “Triumph”). Given the enormous number of clips and constraints on the number of ratings that could feasibly be collected, we opted to collect two ratings of every clip rather than a larger number of ratings of a subset of clips. Raters were asked to describe the emotions felt by the person in the video by selecting all that applied. Annotations were averaged across each video and were conducted in complete blindness to the nature of the eliciting video that the person was reacting to.

### Model comparison

**Figure 4:**
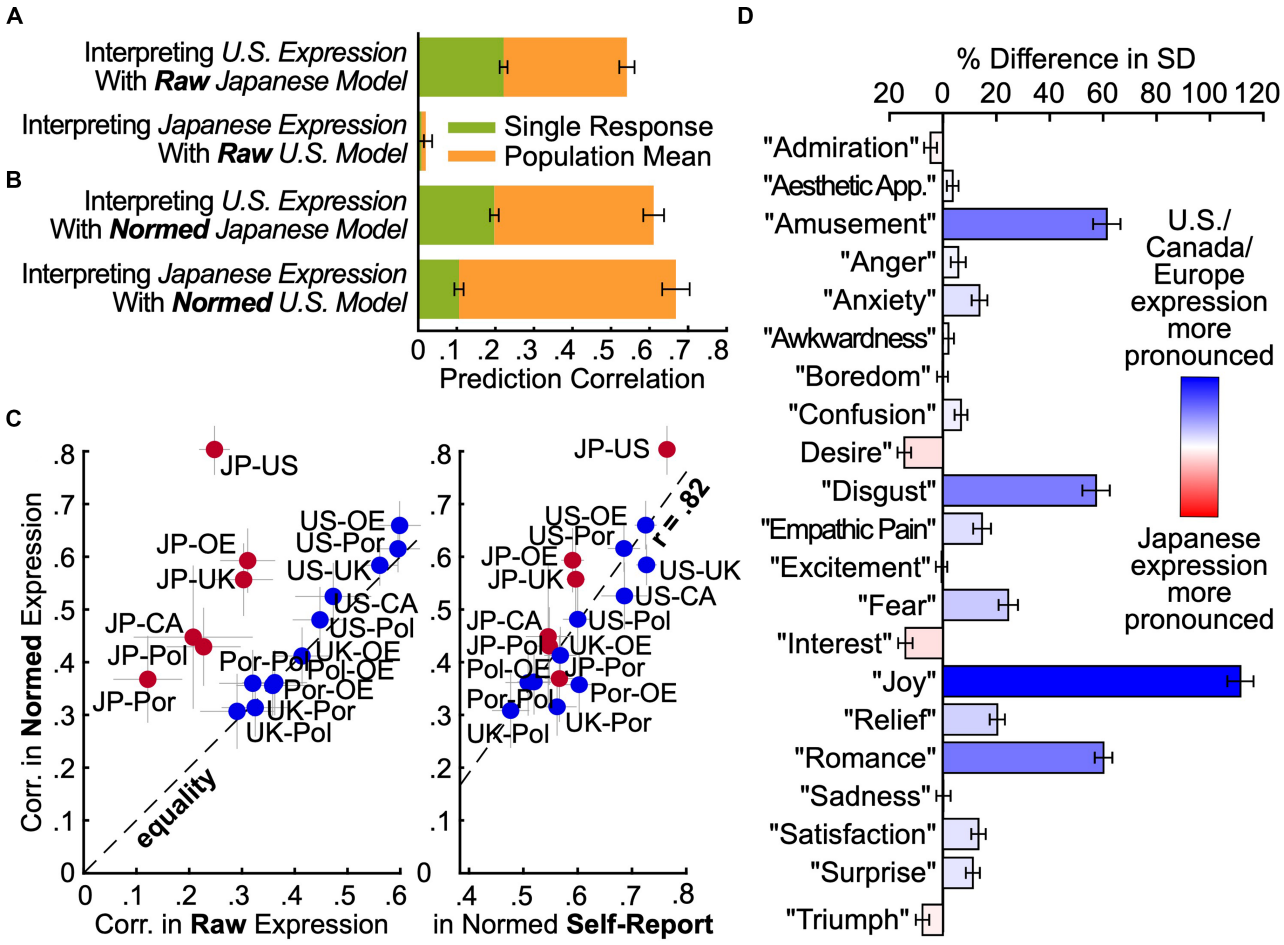
Intensity normalization is required to translate expressions across cultures. **(A)** Culture-insensitive models. To see how well expressions are preserved across the U.S. and Japan, models were trained in one country to predict experience from expression and then applied to responses in the other. Prediction correlations were lower than those of within-country models (Figure 3A), pointing to cultural differences in the mapping from experience to expression. **(B)** Normalization improves prediction by accounting for differences in expression intensity. The same models were trained and tested after normalizing expression intensity within each country (demeaning and dividing by standard deviation). Prediction correlations improved dramatically. Cultural differences in the mapping from experience to expression are largely accounted for by the intensity with which expressions are displayed. **(C)** Country-wise similarity of raw and normalized expression. A model was trained with all data to predict experience from raw expression, then applied to data from each of five individual countries. Predictions were correlated pairwise between countries, before and after normalization. Comparisons between North American/European countries and Japan (red) were dramatically more affected by normalization than those among different North American/European countries (blue). Thus, Japan differs systematically in expression intensity. After normalization, we can see to the right that the correlation in expression between countries closely reflected the correlation in experience (*r* = 0.81, despite noise in each estimate), suggesting that, aside from display tendencies, cultural differences were largely driven by differences in experience rather than differences in what facial expressions mean. CA: Canada, JP: Japan, OE: Other Europe, Pol: Poland, Por: Portugal, UK: United Kingdom, US: United States. **(D)** Differences in intensity of each emotional expression between Japan and the U.S./Canada/Europe. A model was trained with all data to predict experience from raw expression, then standard deviations were computed for each prediction across responses in Japan and the U.S./Canada/Europe. Expressions of many emotions (e.g., “joy,” “disgust”) were much more intense in the North American and European countries than in Japan.

Each model predicting experience from expression was cross-validated using a leave-one-video-out approach. Overall prediction correlations were computed by flattening and correlating the matrices of predicted and actual self-reported emotional experiences projected onto the 21 dimensions. Aside from the analysis in Figure 4B, predicted experiences based on a given participant’s expressive response were always compared with actual self-reported experiences from a different participant, so that we could adjust for explainable variance (see below). Bootstrap resampling of participants was applied to estimate standard errors in prediction correlations.

In applying culture-specific models across cultures an extra calibration was applied to the experience ratings in the culture where the model was evaluated. Specifically, we used linear regression in a leave-one-out fashion to calibrate experience ratings in the culture where the model was evaluated to the experience ratings in the culture where the model was originally fit. This was necessary because a particular English category (e.g., “sadness”) may not translate perfectly to its Japanese counterpart, thus lowering model fit not because the expression has a different meaning but because the labels for the predicted experience rating have subtly different meanings. The information present in the ratings will be weighted somewhat differently after this calibration step, so the maximum prediction correlation may change. Thus, to compare across-culture models to within-culture models (Figure 4A), we trained new within-culture models after performing the same calibration.

### Proportion of explainable variance and sampling error adjustment

When two sets of sample means are correlated, the correlation is biased downward by sampling error. Thus, more ratings would lower the standard error and yield a higher correlation, even though “unbiased” estimates should not be biased by sample size. To correct for this bias, one can divide the sample correlation by the approximate maximum correlation that could be obtained given the sampling error. The maximum attainable correlation is the square root of the proportion of the variance that is not attributable to sampling error (the explainable variance).

Here, we adjust for sampling error at the level of individual responses (self-report ratings and/or facial expression annotations for a given video). In doing so, the maximum attainable correlation is given by the product of the index of reliability for each response, which is equal to the square root of the interrater reliability coefficient. The interrater reliability coefficient was estimated by randomly pairing two responses from separate participants to each video and computing the Pearson correlation across videos. This process was repeated 100 times and the average interrater reliability coefficient was computed.

Prediction correlations for models predicting average experience were adjusted for sampling error in experience by dividing by explainable variance in experience. Prediction correlations for models that used average expression as a predictor were adjusted for sampling error in average expression by dividing by explainable variance in the prediction based on individual expressive responses to the same video, to estimate the maximum prediction correlation that could be achieved with large participant samples.

Explainable variance in the average response is estimated for varying sample sizes in Figure 3. These were estimated using the formula *r*_N_^2^ = ((1-*r*_1_^2^)/(N*r_1_^2^) + 1)^−1^, where *r*_1_^2^ represents the explainable variance of individual-level responses (the square of the interrater reliability coefficient) and *r*_N_^2^ represents the explainable variance of an average of N responses. This formula can be derived from the fact that the signal-to-noise ratio is given by *r*_N_^2^/(1-*r*_N_^2^) and is directly proportional to sample size, such that *r*_N_^2^/(1-*r*_N_^2^) = N*r_1_^2^/(1-*r*_1_^2^). By solving for *r*_N_^2^ we arrive at a simple formula for explainable variance as a function of N.

## Results

When shown emotionally evocative videos – newborn babies laughing, animal carcasses decaying, thunderstorm-filled skies, birds solving puzzles – to what extent do people in different countries report having similar emotional experiences? How many specific kinds of emotion are preserved both in subjective experience across cultures and in the English and Japanese lexica relied on to report upon these experiences? Are cultural commonalities in emotional experience best conceptualized in terms of specific emotions, or movement along valence and arousal dimensions? Answers to these questions are central to contrasting theoretical claims in emotion science (Keltner et al., 2019) and preliminary to investigating whether emotional experiences are expressed in similar facial movements across cultures.

We collected self-reported emotional responses to each of 2,185 evocative videos using surveys in English (averaging 18 responses per video, largely in U.S./Canada and Europe) and Japanese (averaging 7.6 responses per video, all in Japan) (Figure 1). Participants selected from 34 emotion categories (Cowen and Keltner, 2017) and provided 1–100 intensity ratings for each selection (n = 1,220), or rated valence and arousal (*n* = 118) on a 1–9 scale [each derived from Russell, 2003], for a total of 36,606 English-language emotion category responses; 14,856 Japanese-language emotion category responses; 2,802 English-language valence and arousal ratings; and 1,797 Japanese-language valence and arousal ratings. Surveys were translated into Japanese by a team of bilingual researchers (see Methods).

### Similarities in distinct emotional experience across cultures

We first asked how reported emotional experiences were preserved across languages and cultural groups. To do so, we computed correlations between cultural groups in ratings of emotion categories and valence/arousal dimensions, adjusted for within-culture variation (see Methods). As shown in Figures 2A–E, a wide range of reported emotional experiences were well-preserved across the English and Japanese surveys, many with correlations exceeding 0.9 (e.g., “esthetic appreciation”/“美的感動,” “amusement”/“愉快,” “fear”/“恐怖,” “interest”/“興味,” “sadness”/“悲しみ”). As in studies of emotion-related experience, perception, and brain response (Cowen and Keltner, 2017, 2019, 2020; Cowen et al., 2018, 2020; Saarimäki et al., 2018; Cowen A. S. et al., 2019; Horikawa et al., 2020), these specific feeling states were generally better preserved than reports of valence (*r* = 0.74, SE 0.035) and arousal (*r* = −0.065, SE 0.062) of evoked experience, consistent with the latter being higher-order, more culture-specific evaluations (Tsai, 2007; Cowen A. S. et al., 2019; Cowen et al., 2020). The pronounced differences in reported arousal are consistent with other studies comparing English and Japanese speakers (Scherer et al., 1988; Kitayama et al., 2000; Lim, 2016) (see also Supplementary Figure 1).

### Dimensionality in shared emotional experience across cultures

Next, we ask how many distinct kinds of emotional experiences were captured in both the English-language and Japanese survey responses. For instance, “amusement”/“愉快” and “joy”/“喜び” were well-preserved across languages (*r* = 0.92 [SD 0.03], 0.89 [SD 0.05]), but were they used interchangeably? In other words, what is the dimensionality of the (shared) semantic space of emotional experience across the different cultures in this study? As in previous work, we approached this using a multidimensional reliability analysis technique, applying principal preserved components analysis, or PPCA, to determine how many different emotions were evoked in both cultures by distinct videos (Cowen A. S. et al., 2019; Cowen et al., 2020; Demszky et al., 2020) PPCA extracts the distinct patterns of reported emotions that reliably covary across equivalent ratings collected from different groups (see Methods and Video S2 in Supplementary material to understand why PPCA suits this purpose rather than PCA). At least 21 distinct components of subjective experience were preserved across English-and Japanese-language emotion judgments (*q* < 0.005 across all held-out raters, *q* < 0.05 across held-out raters in each culture individually), ForwardStop sequential FDR-corrected (G’Sell et al., 2016) one-tailed Wilcoxon signed-rank test (Wilcoxon, 1945). Thus, at least 21 different kinds of self-reported emotional experience were evoked by the 2,185 videos in different cultural groups and represented in both languages. This converges with our previous work finding 27 dimensions of self-reported emotional experience evoked by videos within a single culture (Cowen and Keltner, 2017) and that reported emotions evoked by music are largely preserved across the U.S. and China (Cowen et al., 2020).

To characterize and interpret the 21 kinds of subjective experience that were captured in both English-language and Japanese responses, we applied factor rotation (varimax) to the 21 significant components extracted using PPCA. This method extracts a more interpretable representation of the 21 components by rotating them onto dimensions that each correspond to as few emotions as possible. Each of the 21 resulting dimensions loaded maximally on a distinct category of emotion reported in response to the 2,185 videos: “Admiration,” “Esthetic Appreciation,” “Amusement,” “Anger,” “Anxiety,” “Awkwardness,” “Boredom,” “Confusion,” “Desire,” “Disgust,” “Empathic Pain,” “Excitement,” “Fear/Horror,” “Interest,” “Joy,” “Relief,” “Romance,” “Sadness,” “Satisfaction,” “Surprise,” and “Triumph” (Figure 2B). These dimensions can therefore be interpreted as 21 distinct kinds of emotional experience that are evoked by similar antecedents in several cultures and are present in both the English and Japanese lexica.

Figures 2C–E visualize the average emotional experiences associated with individual videos and how they are preserved across multiple cultures and languages along the 21 dimensions. We can see visually that the videos are richly diverse in the emotions they evoke, that these emotions are quite similar on average across the English and Japanese surveys (although there was country-level variation within the English survey responses).

We can also see that many videos evoke blends of emotion (e.g., “amusement” and “joy,” “anger” and “fear,” or “esthetic appreciation” and “interest”), consistent with our recent work establishing that the *distribution* of emotions in multidimensional space reveals emotions are heterogeneous and often blend, and do not neatly fall into discrete categories, as is traditionally assumed by Basic Emotion Theory (Ekman and Cordaro, 2011). Instead, dimensions of emotional experience are often bridged by smooth gradients of meaning (Cowen and Keltner, 2017; Cowen et al., 2020) – many instances of Esthetic Appreciation, for example, vary in their typicality or in how they may blend and interact with other states, (states lying somewhere between “esthetic appreciation “and “interest“).

### Variations in emotional experience are signaled in facial expression

We next explored how the 21 kinds of emotional experience uncovered in this study with English-language and Japanese self-reports correspond to patterns of facial movement. The understanding of how facial movements express emotion has been largely oriented around a search for one-to-one mappings between the six “basic” emotions and prototypical facial expressions (Ekman and Friesen, 1971; Elfenbein and Ambady, 2002; Lench et al., 2011; Barrett et al., 2019; Cowen A. et al., 2019). Here we apply discovery-based statistical methods to learn how the 21 dimensions of emotional experience captured in both English-language and Japanese responses may correspond to dozens of distinct patterns of facial movement in thousands of dynamic facial expressions.

More specifically, we collected 45,231 self-recorded reactions to the 2,185 evocative videos. This included, on average, 14.5 reactions to each video from English-language survey participants and 5.6 reactions to each video from Japanese survey participants. The facial expressions in each reaction video were annotated both by human raters in the U.S. (see Discussion for related cultural limitations) and using a deep neural network (DNN). Two human raters annotated each 2 s video segment from each recording with 42 categories of perceived emotion, including the 34 categories used in self-report and 8 others derived from studies of emotions perceived in the face (Cowen and Keltner, 2019; Cordaro et al., 2020), for a total of 648,400 multiple choice ratings (see Methods). Separately, a DNN was used to predict 30 emotion labels attributed to dynamically moving faces [from Cowen and Keltner (2019)], plus “boredom” and “neutral”). The DNN was trained on a separate set of human annotations (by English speakers in India) and relies only on pixels from the face [see Methods and ref. Cowen et al. (2021) for details]. We then, respectively, averaged the human annotations and DNN annotations across each recording (yielding one set of average human annotations and one set of average DNN annotations per reaction). We also computed the maximum of each DNN annotation across each recording. This resulted in a total of 102 annotations per recording: 42 averaged human annotations, 30 averaged DNN annotations, and 30 maximal DNN annotations. In subsequent analyses, we use these annotations as a feature vector characterizing the intensity of facial expressions in each recording along 102 dimensions (Results using human or DNN annotations alone were similar; see Supplementary Figure 2).

To examine how facial expressions related to reported emotional experience, we used the 102 features characterizing facial expressions in reaction to each video to predict the emotional experiences it evoked. We did so by applying linear regression in a leave-one-out fashion across the 2,185 videos (see Methods). To compare specific cultures, we fit separate models in the U.S. and Japan – the two most represented countries in our sample – and examined prediction accuracy at the individual and aggregate levels.

Facial expression predicted reports of emotional experience modestly at the individual level. As indicated in Figure 3A, with single recordings of participants responding to each evocative video, one can predict the average within-culture emotional experience evoked by each video with an overall average correlation of 0.26 (SE 0.01) in the U.S. and 0.14 (SE 0.02) in Japan (between flattened matrices of actual and predicted self-report ratings projected onto the 21 dimensions; see Methods for details). This means that individual facial reactions provide modest insight into what the average person experienced when watching each video [see Video S1 in Supplementary material (https://is.gd/8GsKm8) for example predictions]. This result aligns with recent meta-analyses of small-scale studies of the coherence between facial expression and subjective experience across six well studied emotions (Witkower et al., 2023).

Further analyses explained this was because considerable individual differences in how participants reacted to the 2,185 videos (Lench et al., 2011; Durán et al., 2017). As indicated in Figures 3B, a recording of a single U.S. participant reacting to an evocative video captured, on average, only 14.5% of the variance in expression relevant to predicting average experience across all U.S. participants. Within cultures, individual reactions only modestly inform reaction tendencies across a “culture.” This yields a maximum prediction correlation of 0.38 (√0.145) due to individual variability alone (alternatively, the signal-to-noise ratio would be 0.145/[1–0.145], or 17.0%). Single recordings in Japan were even more variable, capturing just 3.1% of the variance in expression relevant to predicting average experience across Japanese survey participants, corresponding to a maximum prediction correlation of 0.18 (Maximum prediction correlations were computed by taking the product of the index of reliability for each response, which is equal to the square root of the interrater reliability coefficient; see Methods).

Importantly, by averaging expressive responses to each video across enough participants, average reported emotional experience to a given video can be predicted with notable accuracy by facial expression. As the number of participants increases, the overall prediction correlation across emotions, that is the degree to which facial reactions predict self-reports of emotional experience, converges at 0.70 (SE 0.02) in the U.S. and 0.80 (SE 0.05) in Japan using linear models trained to predict reported experience from the expression annotations within each country (Figure 3A). With a model trained on data from all countries, experiences of several emotions (“amusement,” “disgust,” “joy,” “surprise”) can be predicted with correlations of over 0.9 (Figure 3C). At the aggregate level across participants, the emotional expressions evoked by videos are richly informative of subjective experience.

### Cross-cultural convergence in how distinct emotions are expressed in facial action

We assessed the degree of cross-cultural convergence in experience and expression covariation by training models to predict emotional experience in one country from expression in another. Using average facial expression in response to each video in Japan to predict average experience in the U.S., we find that the prediction correlation converges at a very similar value (*r* = 0.65, SE 0.03) to that achieved using U.S. facial expressions (*r* = 0.70, SE 0.02). This finding suggests considerable similarity in the information captured by facial expression across these two cultures. The same is true when predicting average emotional experience in Japan from average expression in the U.S. (*r* = 0.72, SE 0.03) compared to using Japanese expressions (*r* = 0.80, SE 0.05).

Out of the 21 dimensions of emotional experience we uncovered, how many can be differentiated from facial expression alone? Our evidence, which accounts for 72% of the variance in how average expression explains average emotional experience, can provide a lower bound. By applying PPCA between predicted and reported emotional experiences, we found that at least 12 distinct dimensions of emotional experience were predicted from facial expression with significant accuracy (*q* < 0.05 across held-out raters), ForwardStop sequential FDR-corrected (G’Sell et al., 2016) one-tailed Wilcoxon signed-rank test (Wilcoxon, 1945). By examining these 12 dimensions in Figure 3D we can see which experiences mapped onto distinct facial expressions. For instance, “esthetic appreciation” and “romance” were combined into one dimension, suggesting that these feelings are expressed in similar ways. The same is true for “fear” and “anxiety,” “joy” and “relief,” and “sadness” and “anger.” Given that “sadness” and “anger” are assumed to differ substantially in arousal, this contrasts with the predictions of constructivist and core affect approaches, which assume arousal is one of the major features differentiating emotions.

What about the dimensions of emotional experience not distinguished by facial expression? With more evidence, improved machine learning models, and more data, we might eventually uncover more granular distinctions in how people move their faces when they feel emotions, or uncover multimodal associations between the face, voice, and body that explain more consistencies in emotion experience. But for now we conclude that some emotions evoked by video are more readily differentiated from facial expression than others. When we visualize the predictions of average experience along 12 broader dimensions captured by facial expression (Figures 3E,F), we find that they strongly predict reported experience at the aggregate level.

### The relationship between facial muscle movements and emotional experience

**Figure 5:**
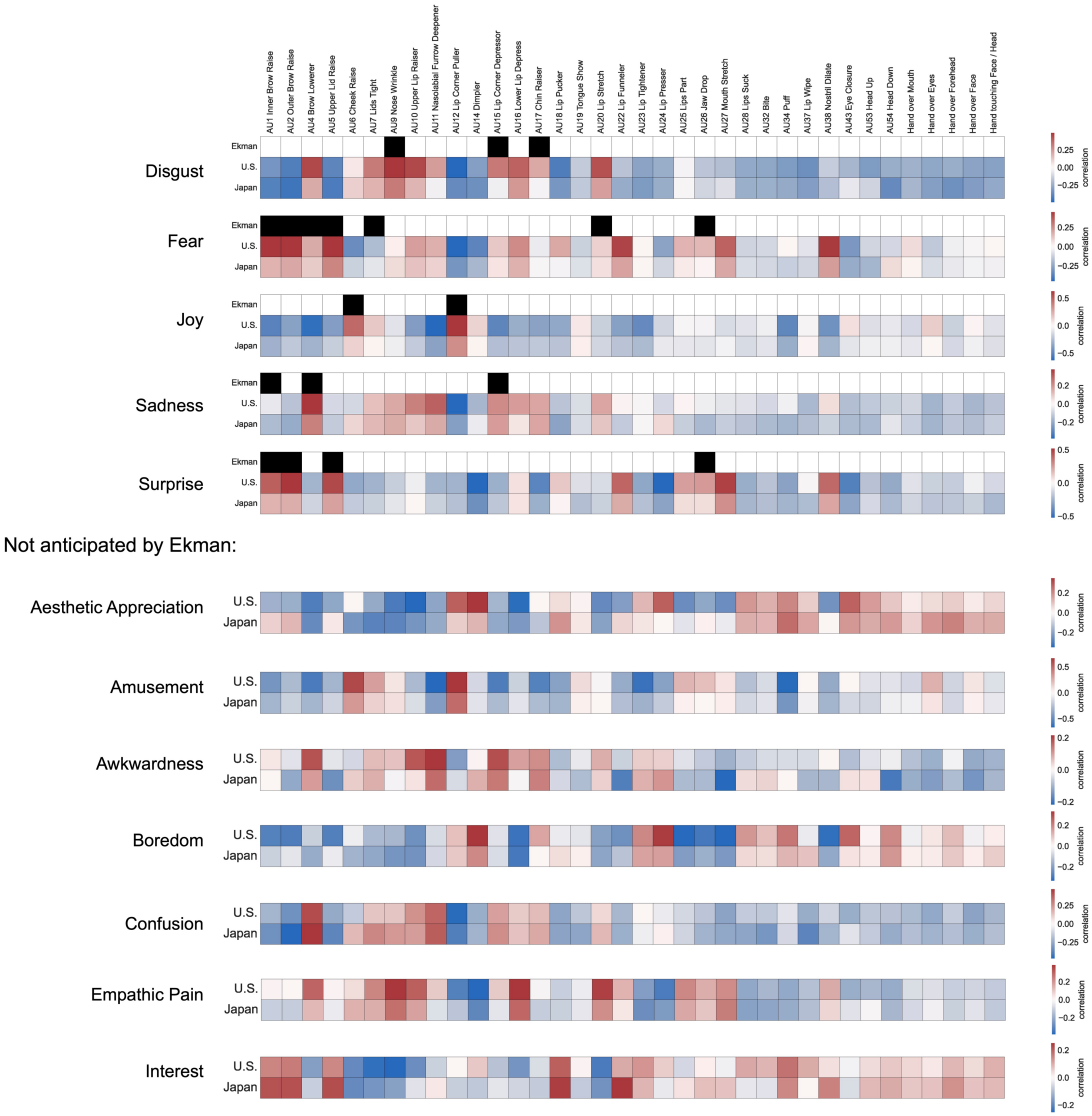
Associations between facial expression dimensions and facial movements. What are the facial movements underlying each dimension of facial expression, and how well do they fit the predictions of past models? To characterize the 12 expression dimensions in terms of underlying facial movements, we correlated the 36 model outputs for each video with their component loadings on each of the 12 dimensions of expression (Figure 3). Each of the top 5 matrices contrasts one of the 12 dimensions in their association with the 36 AUs measured by the FACS DNN in the United States and Japan with the original set of AUs proposed by Ekman to comprise the facial display for a specific kind of emotion. Below, for dimensions without associated AU configurations proposed by Ekman, only the results for the U.S. and Japan are shown.

To characterize the facial actions associated with the 12 dimensions of facial expression that differentiate emotional experiences, we employed an additional DNN that was trained to measure and predict structural aspects of the face. Model outputs were 36 anatomically based dimensions of facial expression drawn from the Facial Action Coding System (FACS), a technique for quantifying the activity of facial muscles and movements of the face and head. The model outputs included 31 facial action units (AUs) and 5 other kinds of face-relevant actions (e.g., “Hand over face”; Figure 5). We adapted our FACS DNN architecture from the FaceNet Inception Resnet v1 model (Schroff et al., 2015), pretrained on the VGGFace2 dataset using transfer learning (Pan and Yang, 2010; Cao et al., 2017) (for further details on model architecture and training, see Methods). We first ran the model on all 45,231 reaction videos. For each second of video, we extracted the face (at a rate of 1 frame per second) using MTCNN (Zhang et al., 2016) and only included the face as input to the model. For each video, we averaged the 36 model outputs across the duration of the video.

What are the facial movements underlying each dimension of facial expression, and how well do they fit the predictions of past models? To characterize the 12 expression dimensions in terms of underlying facial movements, we correlated the 36 model outputs for each video with their component loadings on each of the 12 dimensions of expression. Results are shown in Figure 5, which contrasts each of the 12 dimensions in their association with the 36 AUs measured by the FACS DNN in the United States and Japan with the original set of AUs proposed by Ekman to comprise the facial display for a specific kind of emotion.

How much overlap was there between Japan and the United States in the facial movements that expressed these 12 emotional experiences? As shown in Figure 5, the underlying facial actions associated with each dimension of emotional experience show high agreement between Japan and the United States (the 36 × 12 correlation matrices for the U.S. and Japan were correlated with an *r* of 0.84), with differences mainly emerging in the intensity of specific AUs. In general, the same AUs were associated with each dimension in the U.S. in Japan, with the U.S. showing higher intensity on average. One dimension that showed a different pattern was “Interest”, which Japanese participants expressed with more intensity in AUs associated with both the eyes and mouth (i.e., with AUs such as “Inner Brow Raise”, “Outer Brow Raise”, “Upper Lid Raise”, “Lip Pucker”, “Lip Funneler”). One more notable difference emerged for the “Esthetic Appreciation” dimension, which Japanese participants were more likely to express with their eyes (i.e., with AUs such as “Inner Brow Raiser”, “Outer Brow Raiser”, and “Upper Lid Raiser”).

Seven of the data-driven dimensions which emerged in our dataset were not anticipated by Ekman: “Esthetic Appreciation”, “Amusement”, “Awkwardness”, “Boredom”, “Confusion”, “Empathic Pain”, and “Interest”. These results highlight how automated approaches to measuring and annotating nuanced facial actions contrast with the long-held assumptions of the field, which tend to be low-dimensional, discrete (i.e., binary), and determined *a priori*. These findings reveal more subtle associations between facial actions and facial expressions of emotion, providing the foundation for a more thorough investigation of the underlying anatomical and muscular basis of the dimensions of facial expression, which will be an important task for future research.

### Facial expression across cultures: common meaning but varying display tendencies

We next asked how the expression of emotion differs between countries. Previously, we found that similar information about emotional experience was carried by facial expression in the U.S. and Japan (Figure 3A), but it could have been carried by different patterns of facial movement. Did U.S. and Japanese participants move their faces in similar ways when they felt similar emotions (Barrett et al., 2019; Cowen A. et al., 2019)? To answer this question, we trained models to predict experience from expression in one country and applied them to the other country (Figure 4A). The performance of these models indicates how well the mapping from facial movements to emotional experiences translates across the U.S. and Japan. We found that a model fit to Japanese responses and applied to U.S. expressions did a poorer job of predicting U.S. experience (*r* = 0.54, SE 0.02) than the model that was trained and evaluated within the U.S. (*r* = 0.70, SE 0.02). A model fit to U.S. responses and then applied to Japanese expressions did a far poorer job of predicting Japanese experience (*r* = 0.01, SE 0.03) than the model that was trained and evaluated within Japan (*r* = 0.80, SE 0.05). In other words, there are systematic differences in how people in the U.S. and Japan express emotion with their faces, requiring translation across cultures.

All emotion theories predict variations in emotional expression across cultures, but they posit different kinds of variation. According to some theories, expressions can profoundly differ in meaning across cultures and contexts – the same facial movements can mean very different things (Barrett et al., 2019). Other theories hold that facial expressions have similar meanings across cultures but different norms regulating their use, especially the intensity with which different emotions are expressed (Ekman et al., 1987; Matsumoto and Ekman, 1989). These norms are generally referred to as “dialects” (Elfenbein et al., 2007) or “display rules” (Ekman et al., 1987), but here, we use the term “display tendencies” to more clearly acknowledge individual variation. To what extent are U.S. and Japanese expressions fundamentally different in meaning, and to what extent are they merely different in terms of display tendencies, such as the intensity of the facial movements with which different expressions tend to be displayed (Ekman et al., 1987; Matsumoto and Ekman, 1989)?

**Figure 6:**
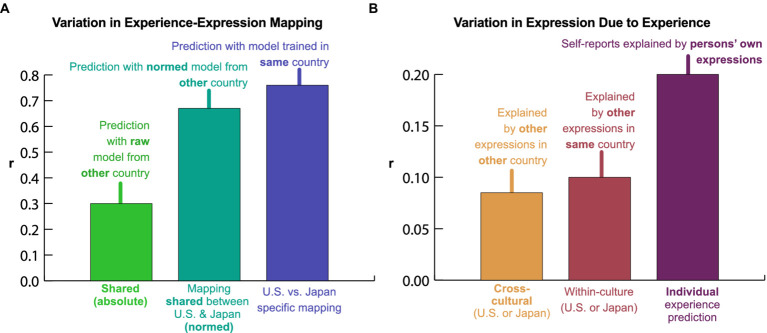
Individual, cultural, and universal variation in expressive response to videos in English-language and Japanese survey participants. **(A)** Intensity normalization largely accounts for variation in the meaning of expressions across cultures. Percentage variance explained by models trained within the same versus other culture (summarizing results in Figures 5A,B and a within-culture comparison model; see Methods: Model Comparison). Prediction correlations from each model were averaged across cultures and squared to compute explained variance. Just 16% of the variance in experience that was explained by within-culture models was explained by models trained in one culture and then applied to the other. However, after intensity normalization, 81% of the variance was explained. Thus, the meaning of expressions is largely preserved across cultures once intensity differences are accounted for. **(B)** Individual variability in emotional experience accounts for the bulk of the systematic variation captured in expression. Figure 1A is concerned with the mapping from experience to expression. It ignores variations in what different people experience when they view the same videos (by predicting experience from expression within culture and adjusting for sampling error across individuals). Here, we examine the degree to which expressive behavior varied as a function of individual experience by applying a single model trained on all data to individual-level expressive responses. To measure how individual, cultural, and universal variation in experience affected expression, we compared how well individuals’ self-reported experiences could be explained by their own expressions versus the expressions of individuals from within the same culture and those of individuals from the other culture. 19% of the individual-level variance in experience explained by expression was shared by individuals in the other culture, 11% was culture-specific, and the remaining 70% corresponded to individuals’ unique emotional experiences.

To answer this question, we again trained models to predict experience from expression in one culture and applied them to the other culture, but we first normalized the 102 features characterizing intensity of facial movements within each culture (subtracted the mean and divided by the standard deviation). This adjusts for cultural variation in the intensity of each pattern of expression. We found that this simple procedure eliminated much of the cultural variation that we had previously observed (Figure 4B). A model trained on normalized Japanese expressions and applied to normalized U.S. expressions predicted U.S. experience more accurately (*r* = 0.61, SE 0.03) than the non-normalized model (*r* = 0.54, SE 0.02). A model trained on normalized U.S. expressions and applied to normalized Japanese expressions predicted Japanese experience far more accurately (*r* = 0.67, SE 0.04) than the non-normalized model (*r* = 0.01, SE 0.03). Based on the performance of these models relative to that of models fit and evaluated within the same culture, we conclude that most of the cultural variation in how expressions map to emotional experience can be attributed to differences in intensity (Figure 6A). Without accounting for intensity differences, only 16% of the variation in the meaning of facial expressions was shared between the U.S. and Japan. After accounting for intensity differences, a full 81% of the variation in the meaning of facial expressions was shared across cultures. A PPCA analysis revealed that this shared variance was carried by at least 11 distinct preserved dimensions; *p* ≤ 0.016, *q* < 0.05 across held-out raters, ForwardStop sequential FDR-corrected (G’Sell et al., 2016) one-tailed Wilcoxon signed-rank test (Wilcoxon, 1945; see Supplementary Figure 3).

Are these differences in expression intensity unique to comparisons between the U.S. and Japan, or can they also be found among different countries in North America and Europe? To answer this question with precision (in countries from which we collected less data than the U.S./Japan), we trained a combined model on all survey responses and applied it to expressive responses in each country (in a leave-one-stimulus-out fashion). We then correlated the model predictions across countries before and after normalizing the predictions (de-meaning and dividing by the standard deviation). Note that the model predictions are linearly dependent on the facial expression annotations. Thus, if the relative intensity of different patterns of facial movement is similar in two countries, then normalization will have little to no effect on the correlation in model predictions between the countries. However, if there are systematic differences in expression intensity, then normalization will serve to increase the correlation.

Thus, it is notable that the correlations between the U.S. and Japan are higher than those between the U.S. and other countries from the English-language sample (Figure 2F). While this could be impacted by differences in sample size or other sources of heterogeneity between the English-speaking countries, it is also an indicator of reliable differences in intensity – as shown in Figure 4C, normalization makes virtually no difference when comparing among the U.S., Canada, U.K., Poland, Portugal, and other European countries. However, when comparing any of these countries to Japan, normalization greatly increases the correlation. Clearly, display tendencies are similar across North American and European countries, but different in Japan.

What exactly is the nature of the differences in intensity between expressions in Japan and the North American and European countries? To answer this question, we computed the standard deviation in model predictions within Japan and the U.S./Canada/Europe. This analysis enabled us to compare the average intensity of the facial movements that express each emotion in each group. As expected, the results of this analysis reveal considerable cultural variation in the intensity of certain expressions. As shown in Figure 4D, expressions of “amusement,” “disgust,” “joy,” and “romance” were over 60% more pronounced in the U.S./Canada/Europe. By contrast, only a few expressions were more pronounced in Japan, and none by more than 15%. These findings suggest that many facial expressions in Japan were substantially more nuanced than in North America and Europe (Scherer et al., 1988; Matsumoto and Ekman, 1989; Sato et al., 2019). To ensure that these findings could not be explained by cultural bias in facial expression annotations, we confirmed that the average expressions in the U.S./Canada/Europe also involved substantially more physical movement of the face than those in Japan; see Supplementary Figure 4. We also verified that the annotations were not biased by racial group; see Supplementary Figure 5.

### Individual differences more robustly predict variation in facial expressions than cultural differences

Thus far we have explored how facial expressions vary across cultures in meaning – that is, in the mapping between facial expression and emotional experience (Figure 6A). We have not explored the extent to which differences in expression arise directly from cultural and individual variations in the experiences evoked by the same videos – for instance, whether different people laugh at different things because they have different senses of humor.

To answer this question, we evaluated how well individuals’ reported experiences could be predicted from their own expressions. Note that this analysis does not adjust for sampling error, as it is unknown how reliably individuals reported on their emotions. Given that there were numerous options – selecting from 34 emotions and rating each on a 1–100 scale – it is likely that individual responses would have low test–retest reliability (even if stimuli could be repeated without being remembered). Hence, to gauge the impact of individual and cultural variation in emotional experience on expression, we assessed how well an individual’s self-reported experiences could be explained by their own expressions versus the expressions of random individuals from within the same country and those of random individuals from the other country. Across these comparisons, reliability is held constant, so it is valid to compare raw prediction correlations in relative terms.

We found that expressions were largely driven by individual variation in emotional experience (Figure 6B). Among U.S. and Japan participants, only 24% of the variance in reported experience explained by expression was shared, on average, by individuals from the other country. Another 6% was shared by individuals within the same country. The remaining 70% corresponded to each individual’s unique emotional experiences and how they manifested in facial expression. This partly explains why single recordings of individual participants’ responses to each video predict the population average reported experience with modest accuracy (Figure 3A). However, the question of how accurately expressions predict individual experience remains difficult, given that we cannot estimate the reliability of individual self-report judgments. While further work is needed to investigate how facial expressions vary at the individual level, the present study establishes that – despite high individual variability – reported experience can be accurately inferred from facial expression at the aggregate level.

## Discussion

The thesis that emotional experiences can be inferred from facial movements, so central to theorizing within the field of emotion science (Barrett et al., 2019; Cowen A. et al., 2019; Keltner et al., 2019), has in large part only been investigated in small samples, generally with a predetermined focus on six prototypical expressions (Elfenbein and Ambady, 2002; Lench et al., 2011; Durán et al., 2017). By analyzing thousands of naturalistic reactions to diverse emotion antecedents, here we paint a detailed portrait of how people in different cultures express emotion. In response to 2,185 evocative videos, people in diverse cultures report at least 21 distinct varieties of emotional experience, which are best conceptualized using specific emotion categories (Figure 2). Average experiences can be predicted with remarkable accuracy from expressive responses to each video at the aggregate level (Figure 3). Facial movements largely have common meaning across multiple cultures, but are subject to differing display tendencies, with many facial expressions in Japan being more nuanced than those in North America and Europe (Figures 4, 6A). Expressions also show high individual variability, which partly reflects individual differences in emotional experiences (Figure 6B).

The present findings address competing hypotheses regarding how emotional expressions vary across cultures. Constructivist approaches hold that cultural variation in expression is observed because expressions are shaped by learned emotion concepts, which are assumed to be highly variable across cultures (Hoemann and Feldman Barrett, 2019). By contrast, Semantic Space Theory (SST) and Basic Emotion Theory (BET) hold that cultural variation will be explained in large part by differential intensities in an expression or rules on when to conceal an expression, but that the expression itself – a specific composite set of facial actions – can be conceptualized similarly across cultures (Durán and Fernández-Dols, 2021; Keltner et al., 2022; Witkower et al., 2023). Our findings more strongly support the predictions of SST and BET in that differences between cultures were in large part driven by variations in the intensity with which different expressions were displayed, not differences in which particular facial movements make up each expression (Figures 5, 6B). With intensity normalized, the mapping from expression to experience was largely preserved across Japan and the U.S. (Figure 6A). Across these cultural groups, facial expressions are subject to differing display tendencies, but have similar meaning.

The findings of the present investigation also speak to theoretical debates regarding how experiences and expressions of emotion are best conceptualized. In BET, specific feelings such as “contempt” and “fear” are more primary in the experience and expression of emotion (Barrett, 2006; Keltner et al., 2019; Cowen et al., 2021), but these states are typically assumed to organize into a 5–10 discrete clusters. Alternative approaches mainly prioritize appraisals of valence and arousal, holding that the underlying representation and conceptualization of emotion will be differentially explained by a linear combination of these broad dimensions (Scherer et al., 1988; Scherer and Wallbott, 1994; Gendron et al., 2018; Barrett et al., 2019).

Our findings support the predictions of SST: Specific emotions (e.g., “awe,” “guilt,” “fear”) structure emotion-related experiences and behavior and have distinct expressions that are shared across cultures, but this space is high-dimensional, including nuanced states such as “Empathic Pain” and “Esthetic Appreciation.” Due to the large degree of individual variability we found, our findings do not support formulations of BET that assume emotions are organized into discrete categories or that facial movements are diagnostic of underlying emotional states (i.e., a one-to-one mapping between experience and expression), and we once again found that dimensions varied along continuous gradients of meaning, not forming clusters. Feelings such as “sadness” and “amusement” were better preserved in self-reported experience across cultures than evaluations of valence and arousal, in contrast with the predictions of constructivist and core affect approaches. These feelings were in turn associated with concurrent facial expressions, which were largely preserved across cultures in meaning and underlying facial movements (Figures 5, 6A; Supplementary Figure 3) and conveyed not two but at least 12 dimensions of emotional experience (Figures 3D–F).

This adds to a growing body of work from the SST framework mapping the specific emotions represented in many different modalities such as self-report, responses to music, and artistic depictions. How do the 21 dimensions of emotional experience and 12 dimensions of facial expression compare to these other data-driven semantic spaces of emotion? The dimensionality of semantic spaces uncovered with this framework are constrained by the number of distinct patterned emotional responses that are picked up on in a given modality and investigation. However we do note that the 21 dimensions of emotional experience largely correspond to those uncovered in previous investigations of emotional experience (Cowen and Keltner, 2017), and the 12 emotions of emotional expression are largely consistent with other semantic spaces of expression (Cowen et al., 2020). It is worth noting that the emotions from earlier smaller taxonomies (i.e., the “basic 6”) were also preserved in our spaces of experience and expression. For further discussion, see our recent synthesis of this research suggesting 18 candidates for universal emotions (Cowen and Keltner, 2021).

This study had several limitations. To characterize facial expressions in several of our analyses, we used human and DNN annotations that were based on English-language categorizations of perceived emotion. Although these annotations were broadly predictive of aggregate emotional experience in both English-speaking countries and Japan (Figures 3A, 4B), it is still important to note that they were constrained by the culture-specific knowledge of the raters used for manual annotation and for training the DNN (see Methods). For instance, Japanese categorizations of perceived emotion could potentially reveal additional dimensions of emotional response, potentially with different display tendencies. This is particularly true given that our approach assumes an intuitive mapping between Japanese-language translations of English emotion categories, but in some cases there may by nuance in usage that were not captured here.

However, we note that this would have no impact on our main finding that facial expressions predict *at least* 12 dimensions of experience. For example, if we had also collected Japanese-language-based DNN annotations of the responses and concatenated them with the English-language-based ones, the 30 English-language-based DNN outputs (which depend only on facial movement) would be the same; they would still predict the same 12 dimensions of experience. The additional Japanese-language-based annotations, capturing additional facial movements, could only predict additional dimensions. Such research could build on our findings and provide further insight into how people in different cultural groups express (and perceive) emotion. Relatedly, the design of our study and analytic approach was taken to maximize our ability to measure the convergence in emotional expressions between cultures. It is possible that additional dimensions could be uncovered in which cultures systematically diverge, had we taken that approach.

On a related note, it is worth acknowledging that cross-cultural similarities in emotion-related behavior may be explained in part by cultural globalization. To further examine human universals in expression, it would be fruitful to apply similar methods in remote, small-scale cultures, although such work would likely be more constrained in scale. Finally, we relied on videos to evoke emotion, but more personal forms of emotion elicitation (e.g., social interaction) would likely reveal further dimensions of emotional expression (Cowen et al., 2021).

The present study focused on facial expressions formed in isolation, but our everyday understanding of facial expression is sensitive to context. An important direction for future work will be to incorporate vocal expression, hand gestures, and other sources of extra-facial information to further characterize the complex ways in which people across cultures express emotion and to better understand how these expressions relate to those described here from the face. More broadly, social context and the eliciting stimuli matter. Here, participants viewed evocative videos alone, which could introduce differences in facial movements compared to more direct, firsthand emotional experiences, and although they were encouraged to use their face expressively, emotional expression would likely vary in the presence of others (Jakobs et al., 2001), an important topic for further study. Participants were also necessarily aware of the presence of observation and were explicitly instructed to react to the videos expressively, making it difficult to state whether a given reaction is truly “spontaneous” or unposed, and possibly opening the results to the influence of observer or desirability effects (i.e., participants could have been forming the expressions they felt they “should” form in response to the evocative videos). Moreover, there was no way to test whether any potential differences in the spontaneity or naturalness of expressions differed between cultures. Although our paradigm allows for measurement of much more unconstrained facial expressions than in previous work, further research is required to further characterize the nuanced facial expressions made in and out of social contexts, and how these differences could covary with cultural differences.

The defining moments of our lives – romance, friendship formation, loss, discovery, betrayal – are rife with emotional expression. Yet our scientific understanding of the meaning of expressions has been limited by methods and theories which prioritize a narrow focus on whether six emotions map to prototypical facial movements. Open-ended methods and large-scale evidence paint a more comprehensive picture – a detailed portrait of the complex ways in which people move their faces in response to thousands of emotionally evocative scenes. Across diverse cultural groups in North America, Europe, and Japan, we find that facial expressions reflect a broad array of specific feelings, are similar in meaning, and are subject to varying display tendencies.

## Data availability statement

The raw data supporting the conclusions of this article will be made available by the authors, without undue reservation.

## Ethics statement

The studies involving humans were approved by University of California, Berkeley Institutional Review Board. The studies were conducted in accordance with the local legislation and institutional requirements. The participants provided their written informed consent to participate in this study. Written informed consent was obtained from the individual(s) for the publication of any potentially identifiable images or data included in this article.

## Author contributions

AC: Writing – original draft, Writing – review & editing, Conceptualization, Data curation, Formal analysis, Funding acquisition, Investigation, Methodology, Project administration, Resources, Software, Supervision, Validation, Visualization. JB: Formal analysis, Investigation, Methodology, Visualization, Writing – original draft, Writing – review & editing. GP: Conceptualization, Data curation, Investigation, Software, Writing – review & editing. MT: Writing – review & editing, Data curation, Investigation, Methodology. YK: Data curation, Investigation, Methodology, Writing – review & editing. VK: Data curation, Investigation, Methodology, Software, Writing – review & editing. KS: Data curation, Investigation, Methodology, Software, Writing – review & editing. BJ: Data curation, Investigation, Methodology, Software, Writing – review & editing. FS: Data curation, Investigation, Methodology, Software, Writing – review & editing. HA: Data curation, Investigation, Methodology, Software, Writing – review & editing. DS: Formal analysis, Software, Writing – review & editing. XF: Data curation, Investigation, Methodology, Software, Writing – review & editing. KM: Formal analysis, Methodology, Software, Writing – review & editing. PT: Writing – review & editing, Software. MO: Writing – review & editing, Software. DK: Funding acquisition, Project administration, Resources, Supervision, Validation, Writing – review & editing.

## Footnotes

^1^https://pytorch.org/docs/stable/generated/torch.nn.AdaptiveAvgPool2d.html
