# Supplementary material for: How emotion is experienced and expressed in multiple cultures: a large-scale experiment across North America, Europe, and Japan
Source: Front Psychol. 2024 Jun 20;15:1350631. doi: 10.3389/fpsyg.2024.1350631 (PMC11223574; doi:10.3389/fpsyg.2024.1350631)
Supplement: Supplementary file 1 [file Data_Sheet_1.pdf]

Supplementary Information for

How emotion is experienced and expressed in multiple cultures: A large-scale experiment across North America, Europe, and Japan

Alan S. Cowen, Jeffrey A. Brooks, Gautam Prasad, Misato Tanaka, Yukiyasu Kamitani, Vladimir Kirilyuk, Krishna Somandepalli, Brendan Jou, Florian Schroff, Hartwig Adam, Disa Sauter, Xia Fang, Kunalan Manokara, Panagiotis Tzirakis, Moses Oh, & Dacher Keltner

Corresponding author: Alan S. Cowen
Email: alan@hume.ai

This PDF file includes:

Supplementary text
Figures S1 to S6
Table S1
Legends for Movies S1 to S2
SI References

Other supplementary materials for this manuscript include the following:

Movie S2

Supplementary text

Correlations between cultural groups. We computed the correlations between ratings of each category across cultural groups (countries or survey languages). To do so, for each rating (emotion category or valence/arousal dimension), we first drew individual ratings at random from each cultural group. One rating or score was drawn for each of the 2185 videos. For instance, when comparing ratings of "disgust", one rating of each video was drawn from each cultural group (each ranging from 0-100). We then computed the correlation in the ratings between the cultural group, across the 2,185 videos. Finally, we divided by the maximum attainable correlation, or noise ceiling, based on within-culture variability of the ratings. As explained further below, the maximum attainable correlation is estimated by computing the geometric mean of the correlations between individual ratings drawn at random from within the same cultural group.

Principal preserved component analysis (PPCA). PPCA extracts shared dimensions that maximize the covariance between two parallel datasets (e.g., emotions ratings). To do so, PPCA first seeks a unit vector α_1 that maximizes the objective function

$$\mathrm{Cov}(X\alpha_1, Y\alpha_1)$$

in which we call α_1 the first principal preserved component. Subsequent components are obtained by seeking additional unit vectors α_i that maximize the objective function $\mathrm{Cov}(X\alpha_i, Y\alpha_i)$ subject to the constraint that α_1 is orthogonal to the previous components, $\alpha_1,\ldots, \alpha_{i-1}$.

In the special case that X = Y, PPCA is equivalent to PCA, given that the latter method maximizes the objective function

$$\mathrm{Var}(X\alpha_i) = \mathrm{Cov}(X\alpha_i, X\alpha_i)$$

(substituting another X for Y in $\mathrm{Cov}[X\alpha_1, Y\alpha_1]$). See Video S2 for illustration of why the PCA objective is ill-suited to our aims. Also note the similarity to the PLSC objective, which seeks to find two separate bases and ß to maximize $\mathrm{Cov}(X\alpha_i, Y\beta_i)$, as well as the CCA objective, which seeks to maximize $\mathrm{Corr}(X\alpha_i, Y\beta_i)$. However, given our aim of finding *preserved* dimensions across ratings (between cultures, or predicted vs. reported), PPCA derives only one basis, α, that applies to both datasets. (In PPCA, therefore, the data matrices must be commensurate: observations in both datasets must be of the same dimension; i.e. the number of rows in X and Y must be equal.)

To solve the PPCA objective and find an α_1 we apply eigendecomposition to the addition of the cross-covariance matrix between datasets and its transpose: $\mathrm{Cov}(X,Y)/2 + \mathrm{Cov}(Y,X)/2$. We claim that the principal eigenvector of this symmetric matrix maximizes $\mathrm{Cov}(X\alpha_1, Y\alpha_1)$. To derive this, first recall a general property of cross-covariance, $\mathrm{Cov}(Xa, Yb) = b^T\mathrm{Cov}(X, Y)a$. Thus,

$$\mathrm{Cov}(X\alpha_1, Y\alpha_1) = \alpha_1^T\mathrm{Cov}(X, Y)\,\alpha_1 \qquad \text{(Property 1)}$$

In addition, because both $X\alpha_1$ and $Y\alpha_1$ are vectors, $\mathrm{Cov}(X\alpha_1, Y\alpha_1) = \mathrm{Cov}(Y\alpha_1, X\alpha_1)$. Thus,

$$\mathrm{Cov}(X\alpha_1, Y\alpha_1) = \mathrm{Cov}(X\alpha_1, Y\alpha_1)/2 + \mathrm{Cov}(Y\alpha_1, X\alpha_1)/2 \qquad \text{(Property 2)}$$

Combining these two properties, we can see that

$$\mathrm{Cov}(X\alpha_1, Y\alpha_1) = \mathrm{Cov}(X\alpha_1, Y\alpha_1)/2 + \mathrm{Cov}(Y\alpha_1, X\alpha_1)/2 \qquad \text{(By property 2)}$$

$$= \alpha_1^T\mathrm{Cov}(X, Y)\,\alpha_1/2 + \alpha_1^T\mathrm{Cov}(Y, X)\,\alpha_1/2 \qquad \text{(By property 1)}$$

$= \alpha_1^T[\mathrm{Cov}(X , Y)/2 + \mathrm{Cov}(Y , X)/2]\ \alpha_1$

Now, letting R = [Cov(X,Y)/2 + Cov(Y,X)/2], we see that maximizing $\alpha_1^T R \alpha_1$ is equivalent to maximizing Cov($X\alpha_1$, $Y\alpha_1$), the originally stated PPCA objective. (Note that if X = Y, we are applying eigendecomposition to Var[$X\alpha_i$] = Cov[$X\alpha_i$, $X\alpha_i$], which performs PCA.)

Finally, the min-max theorem dictates that the principal eigenvector of R maximizes $\alpha_1^T R \alpha_1$ subject to α_1 being a unit vector ($||\alpha_1||=1$)

We have thus found a unit vector α_1 that maximizes Cov($X\alpha_1$, $Y\alpha_1$)—the covariance between the projections of X and Y projected onto the first component. Based on the min-max theorem, subsequent eigenvectors α_i will maximize Cov($X\alpha_i$, $Y\alpha_i$) subject to their orthogonality with previous components α_1 through α_{i-1} and to each α_i also being a unit vector ($|\alpha_i|=1$).

We note that the min-max theorem also provides that the last eigenvector, α_n, will minimize Cov($X\alpha_n$, $Y\alpha_n$), equivalent to maximizing -Cov($X\alpha_n$, $Y\alpha_n$). Hence, if there are dimensions of negative covariance between the two datasets, then some eigenvectors will maximize the negative covariance.

With respect to the corresponding eigenvalues, each eigenvalue λ_i will be equal to Cov($X\alpha_i$, $Y\alpha_i$). To see this, note that:

$$[\mathrm{Cov}(X , Y)/2 + \mathrm{Cov}(Y , X)/2]\ \alpha_i = \lambda_i\ \alpha_i \qquad \text{(Eigenvalue equation)}$$

$$\alpha_i^T\ [\mathrm{Cov}(X , Y)/2 + \mathrm{Cov}(Y , X)/2]\ \alpha_i = \alpha_i^T\ \lambda_i\ \alpha_i$$

$$\mathrm{Cov}(X\alpha_i, Y\alpha_i) = \lambda_i\ \alpha_i^T\alpha_i \qquad \text{(By property 1)}$$

Now $\alpha_i^T\alpha_i = 1$ because the α_i are orthonormal. Hence,

$$\mathrm{Cov}(X\alpha_i, Y\alpha_i) = \lambda_i$$

This also entails that there will be negative eigenvalues corresponding to negative covariance.

We performed PPCA between the averaged self-report ratings from each culture (Fig. 2) or between the averaged self-report ratings across cultures and the predicted self-report ratings from our model (Fig. 3D-F). To ascertain the number of significant dimensions of covariance, we performed a leave-one-subject-out analysis, in which PPCA was iteratively performed on data from all but one participant and then the held-out ratings were projected onto the extracted dimensions and correlated. Partial Spearman correlations were used, controlling for projections onto previous dimensions, to account for possible curvilinear relationships. Wilcoxon signed-rank tests were then applied to the correlations for held out raters to test each dimension for significance.

Facial expression DNN architecture. Face-based visual features were extracted using layers from the NN2 FaceNet architecture to characterize mid-level attributes involved in early stages of face perception. These layers consisted of an Inception (5a) block with a 7x7 feature map comprising 1,024 channels, which was fed into a 7x7 average pooling layer, generating a 1,024 dimensional vector representing face image features within a given frame of the video. The resulting features were then fed into two long short-term memory (LSTM) layers, each with 64 recurrent cells, to capture the dependence of facial expression recognition on temporally unfolding patterns of facial movement. Finally, the output of the last LSTM layer was fed into a mixture-of-experts model (two experts, plus a dummy expert). A cross entropy loss with a sigmoid activation function was used for the final layer with thirty nodes.

Facial expression DNN Training. The DNN was trained on a total of 342,546 ratings of 247,292 video clips of facial expressions independently gathered on YouTube. Clips were extracted from videos which were manually collected by raters, who were instructed to conduct a broad search for videos likely to contain emotional expressions. The facial expression clips were then rated by English speakers in India. The task was to select all facial expression categories that applied to each face.

FACS DNN architecture. We utilized layers from the FaceNet Inception Resnet v1 model, pretrained on the VGGFace2 dataset via transfer learning. We froze all layers up until the last convolutional block and unfroze the last convolutional block. On top of this architecture we added the following fresh untrained layers: 2D adaptive average pool (output_size = 1; https://pytorch.org/docs/stable/generated/torch.nn.AdaptiveAvgPool2d.html), followed by a dropout layer (p = 0.6). The features were then flattened and fed to a linear (1790 in features → 512 out features) layer, followed by Batchnorm1d (eps=0.001, momentum = 0.1, affine = True) and a final linear layer (512 in features → 48 * 4 out features).

FACS DNN training. The DNN was trained on 467,566 static images of facial expressions collected for a separate study, in which web-based participants used a computer webcam to photograph themselves mimicking facial expressions. 1,500 of the original images were coded on the presence or absence of 48 Action Units (AUs) by two certified expert FACS coders.

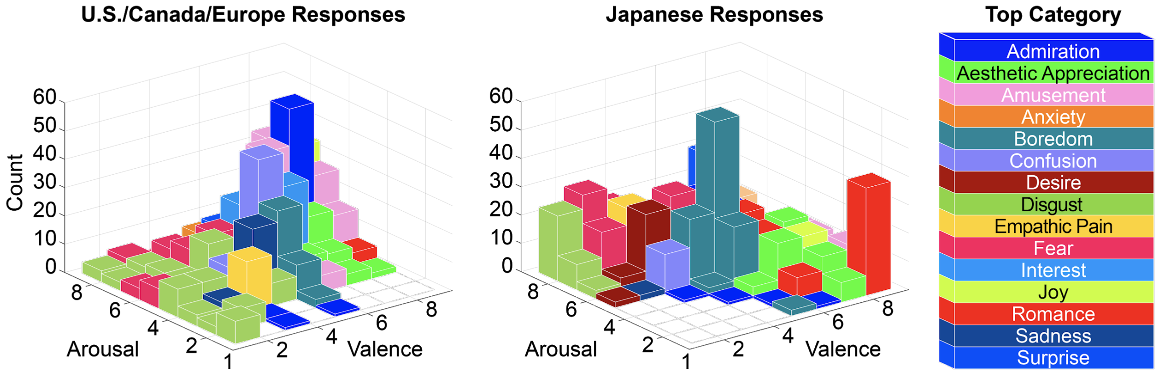

Fig. S1. Distribution of valence and arousal ratings in U.S./Canada/Europe and Japan. Histogram of valence and arousal ratings in each cultural group, colored according to the highest average intensity emotion category in judgments of videos represented by each bin (within the culture). Close inspection of these histograms reveals why English-language and Japanese arousal ratings had little to no correlation. For example, videos that evoked "disgust" were often assigned low arousal ratings in the U.S./Canada/Europe but had almost uniformly high arousal ratings in Japan (whereas videos evoking "fear" were assigned high arousal ratings in both cultures). Videos that evoked "romance", "aesthetic appreciation", and most other positive emotions were assigned low arousal ratings in Japan. Generally, negative emotions were higher arousal in Japan than in the U.S./Canada/Europe, and positive emotions were higher arousal in U.S./Canada/Europe than in Japan. These findings are consistent with research on affect valuation in so-called "Western" and "Eastern" cultures27 and more focused cultural comparisons, which consistently find that high arousal positive emotions are more common in North American and European countries than in Japan20–22,34.

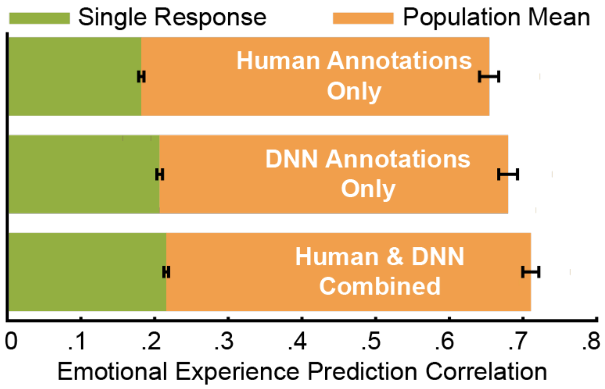

Fig. S2. Human and DNN annotations alone each predicted emotional experience with only slightly lower accuracy than the combined model. Models were trained using linear regression to predict average experience in response to each of the 2185 videos from average expression annotations combined across cultures. Models based on human or DNN annotations alone performed only slightly worse than the full model, suggesting that human and DNN annotations largely overlap in the information they capture. Note that performance of the human annotation model is not adjusted for sampling error and could potentially improve with additional annotations.

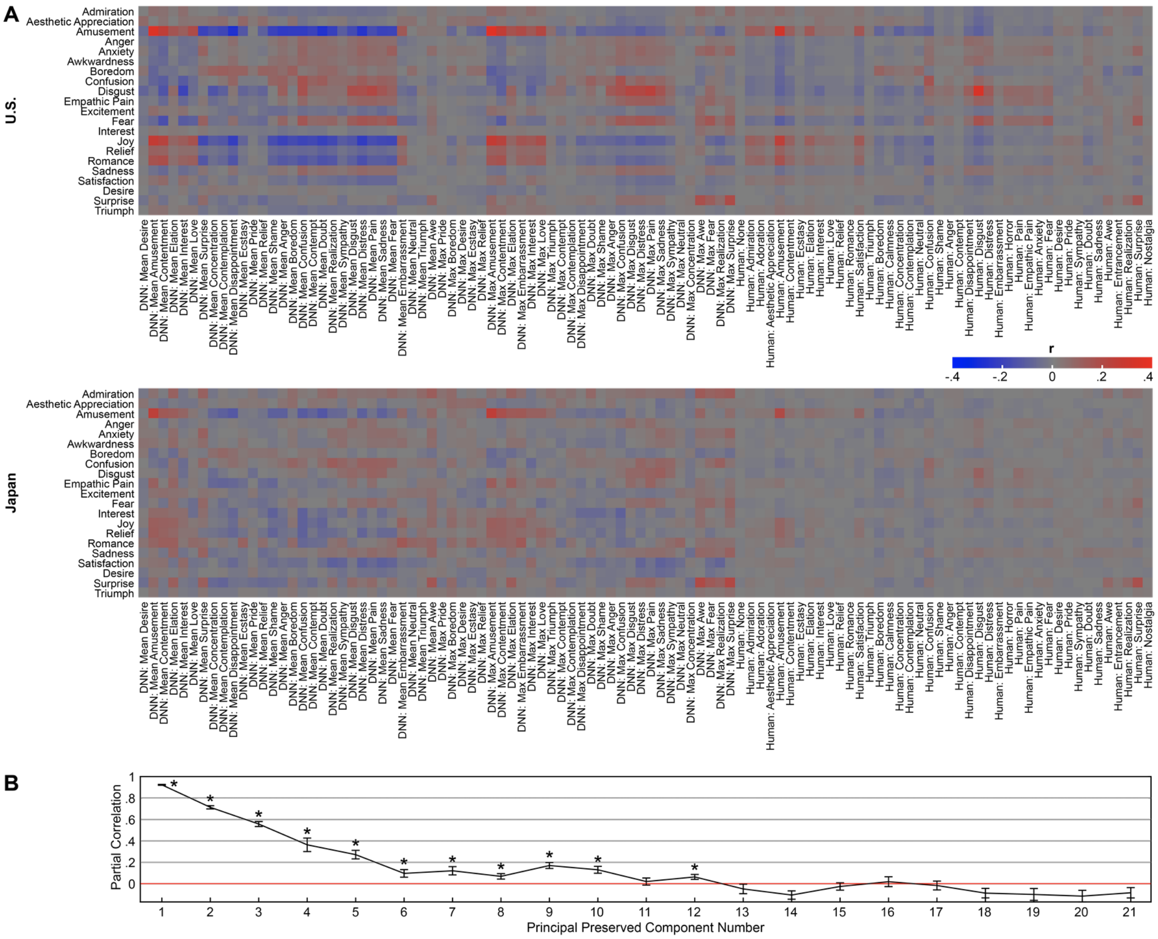

Fig. S3. Experience-expression association across the U.S. and Japan. ***A. Raw individual-level correlations between 21 dimensions of emotional experience and 102 DNN and human expression annotations in each country.*** Annotations are ordered by maximally-correlated dimension of emotional experience. Correlations are similar in the two cultural groups, albeit more nuanced in Japan. ***B. Dimensions required to explain preserved experience-expression correlations across cultures.*** PPCA was applied to the 102x21 matrices of correlations between cultures. This analysis was performed in a 13-fold cross-validated fashion across U.S. participants, iteratively leaving out data from 77 of the 1001 participants that contributed both experience judgments and self-recorded expressions. Correlations were separately computed between experience and expression in the held-in and held-out data. PPCA was then performed between the held-in U.S. correlations and the correlations in Japan. Finally, the held-out U.S. correlations and the Japanese correlations were projected onto the extracted components and correlated across cultures (partial Spearman correlations, controlling for previous dimensions). Eleven dimensions were significant (*p≤.016, q<0.05 across folds, ForwardStop sequential FDR-corrected29 one-tailed Wilcoxon signed-rank test30). Japanese data were not subdivided during cross-validation, because the data were more limited, but cross-validation within the U.S. responses is sufficient to guarantee independence. With additional data, it is likely that further dimensions would be discovered.

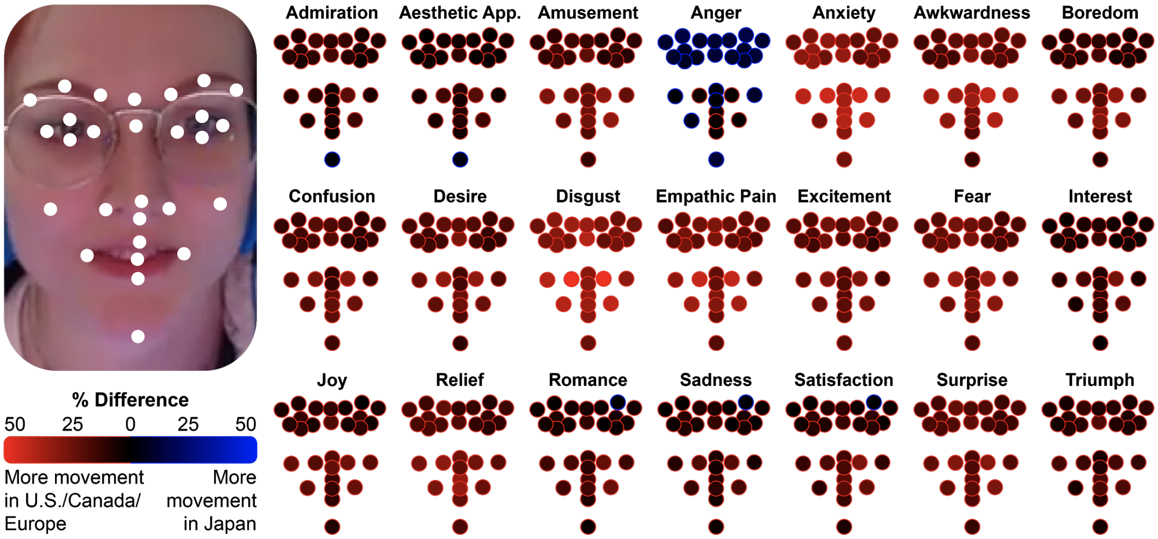

Fig. S4. Differences in average facial movement between U.S./Canada/Europe and Japanese participants. To measure physical movement of the face, we used a face tracking algorithm to locate 29 facial landmarks in each of the 45,231 reaction recordings on a frame-by-frame basis (2 frames per second). Across each reaction, we then computed the standard deviation in the average distance between each facial landmark and the three other facial landmarks to which it was most often positioned closest. We divided by the average distance in the video, yielding proportional measures of local facial movement. These measures were averaged within each cultural group across reactions to videos that most evoked each of the 21 dimensions of emotional experience. Facial movements in response to most videos were more physically pronounced in U.S./Canada/Europe participants, on average (the only minor exception being responses to "anger"-evoking videos). The difference is present in every region of the face, especially near the cheeks, mouth, and nose, where physical facial movements in response to "disgust"-evoking stimuli were more than 50% more pronounced in the U.S./Canada/Europe than in Japan. These findings confirm that the greater intensity we observe in average U.S./Canada/Europe expressions (Fig. 4C) cannot be explained by a cultural bias in facial expression annotations.

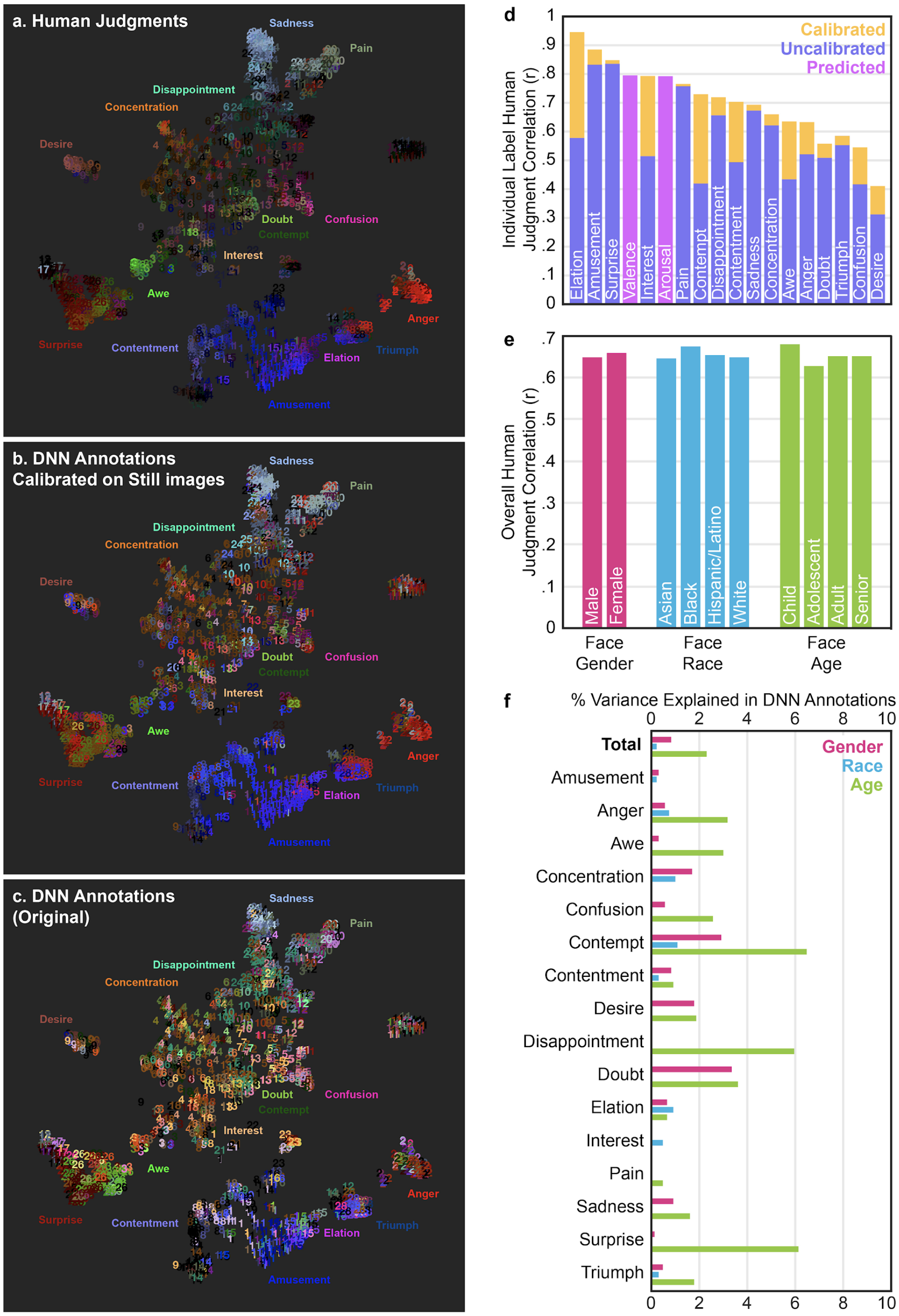

Fig. S5. The expression DNN predicts human judgments and is largely invariant to demographics (from Cowen et al., Nature). ***a-c. Accuracy of the expression DNN in emulating human judgments.*** Human judgments (a) and the expression DNN annotations (b-c) have been projected onto a map of 1456 facial expressions adapted from Cowen & Keltner13. Human judgments and the expression DNN annotations are represented using colors, according to the color scheme used in Cowen & Keltner13. Likely because the expression DNN was trained on dynamic faces, it can in some cases make systematic errors in predicting judgments of static faces (c). For example, a number of static faces of “surprise” were more strongly annotated by the expression DNN as “awe” (c, bottom left), likely because dynamic faces that convey “surprise” are distinguished in part by dynamic movement. This problem is mitigated when the expression DNN is calibrated for still images (B). To calibrate the DNN, multiple linear regression is applied in

a leave-one-out-fashion to predict human judgments of the still images from the DNN annotations. After calibration on still images, we can see that the expression DNN annotations are fairly accurate in emulating human judgments (overall r = .69 between calibrated expression DNN annotations and human judgments after adjusting for explainable variance in human judgments***). d. The expression DNN can emulate human judgments of individual emotions and valence/arousal with moderate to high accuracy.*** Individual expression DNN predictions are correlated with human judgments across the 1456 faces. Valence and arousal judgments (also from Cowen & Keltner13) were predicted using multiple linear regression in a leave-one-out fashion from the 16 facial expression DNN annotations. ***e. The expression DNN is reliable for different demographic groups.*** By correlating the expression DNN predictions (calibrated for static images) across subsets of the 1456 faces from Cowen & Keltner13 we can see that the expression DNN is accurate for faces from different demographic groups (adjusted for explainable variance in human judgments). ***f. The expression DNN has little bias across demographic groups.*** To assess demographic bias, the expression DNN annotations of each face were predicted by averaging the expression DNN annotations across all other faces from the same demographic group. The variance in the expression DNN annotations explained by demographic group in this dataset was quite low, even though no effort was originally made to balance expressions in this dataset across demographic groups. Gender explained 0.88% of the total variance, race explained 0.28%, and age explained 2.4%. Results for individual expressions were generally negligible, although age did explain more than 4% of the variance for three expressions—“contempt”, “disappointment”, and “surprise” (max = 6.2% for “surprise”). Note that these numbers only provide a ceiling for the demographic bias, given that explained variance may also derive from systematic associations between expression and demographics in this naturalistic dataset—for example, because elderly people are less often pictured playing sports, they are less likely to be pictured with expressions that occur during sports. We can conclude that the expression DNN is largely unbiased by race and gender, with age possibly having at most a minor influence on certain annotations.

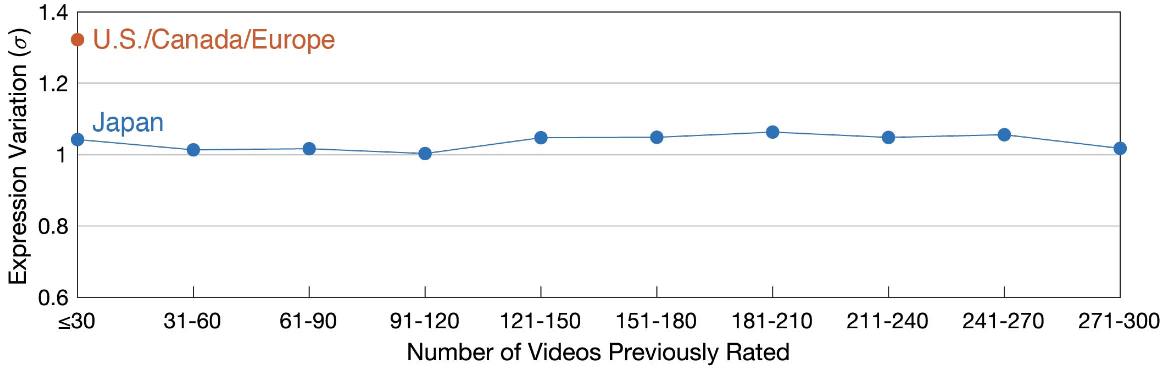

Fig. S6. Expression intensity by number of videos previously rated. Variation in expression was computed as the average standard deviation across expression-based emotion predictions using a linear model trained on all data. Model predictions were first normalized by subtracting the mean across all Japanese responses and dividing by the standard deviation across all Japanese responses. Because Japanese participants responded to many videos, it is critical to note that their expressions did not decline in intensity (operationalized as variation) over time. Variation in expression in the U.S./Canada/Europe is shown for comparison (most English-language survey participants responded to 30 videos or less).

Table S1. Translating English-language emotion terms into Japanese.

English	Japanese	Reference(s)
Admiration	称賛	48
Adoration	崇拝	Bulk search of English-Japanese dictionaries via https://ejje.weblio.jp/. Note that the term "崇拝" is not used in response to "cute" stimuli as is the English "adoration" ("崇拝" is closer to literal "worship"), which explains why ratings were not consistent across cultures (Fig. 2).
Aesthetic appreciation	美的感動	Bulk search of English-Japanese dictionaries via https://ejje.weblio.jp/
Amusement	愉快	49
Anger	怒り	49
Anxiety	不安	50
Awe	畏敬	49
Awkwardness	ばつの悪さ	51
Boredom	退屈	52
Calmness	落ち着き	Based on "落ち着いた気持ち" from [49]
Confusion	困惑	Bulk search of English-Japanese dictionary via https://ejje.weblio.jp/
Contempt	軽蔑	53
Craving	渇望	Bulk search of English-Japanese dictionary via https://ejje.weblio.jp/
Desire	性欲	49
Disappointment	失望	54
Disgust	嫌悪	50
Empathic Pain	痛みの共感	Bulk search of English-Japanese dictionaries via https://ejje.weblio.jp/

Entrancement	没入感	The most direct translations of "entrancement" – "忘我の境地", "恍惚状態", "有頂天", or "狂喜" – are not easily understood as an emotional state, or have a more pleasant connotation. The term "没入感" was selected because it more directly conveys how a video can capture one's awareness.
Envy	妬み	Selected from [54]
Excitement	興奮	[49]
Fear	恐怖	Adapted from "恐れ" from [50]
Guilt	罪悪感	[50]
Horror	戦慄	Both "horror" and "fear" are most directly translated to "恐怖". The term "戦慄" was selected for horror because it is more intense and visceral/physical than "恐怖".
Interest	興味	[49]
Joy	喜び	[49]
Nostalgia	郷愁	[49]
Pride	自尊心	[49]
Relief	安心	[49]
Romance	情愛	Since the most direct translation ("ロマンス") is not easily understood as an emotional state, chose "情愛" (roughly, "affection")
Sadness	悲しみ	[49]
Satisfaction	満足	[49]
Surprise	驚き	[49]
Sympathy	同情	[49]
Triumph	勝利感	Bulk search of English-Japanese dictionaries via https://ejje.weblio.jp/
Subdued (arousal)	気持ちを穏やかにする	Direct translation, confirmed by bulk search of English-Japanese dictionaries via https://ejje.weblio.jp/. Term chosen in part based on neutral valence connotation.

Stimulated (arousal)	刺激的な	Direct translation, confirmed by bulk search of English-Japanese dictionaries via https://ejje.weblio.jp/. Term chosen in part based on neutral valence connotation.
Unpleasant (valence)	不快	Direct translation, confirmed by bulk search of English-Japanese dictionaries via https://ejje.weblio.jp/.
Pleasant (valence)	快	Direct translation, confirmed by bulk search of English-Japanese dictionaries via https://ejje.weblio.jp/.

Movie S1 [https://is.gd/8GsKm8]. **Example reactions, self-report, and predicted emotion for four stimuli.** Reactions on the left side of each stimulus are from the U.S. (stimuli 1, 3, and 4) and U.K. (stimulus 2). Reactions on the right side of each stimulus are from Japan. Self-report ratings have been projected onto the 21 dimensions extracted using PPCA (Fig. 2) for comparison across cultures/languages. Emotions are predicted via models trained and evaluated on data specific to each survey language (English or Japanese). Explore more reactions at https://tinyurl.com/yywa7kjf.

Movie S2 (separate file). Illustrating why principal component analysis (PCA) or factor analytic methods are not designed to account for the reliability of judgments of individual features. Each scatterplot represents hypothetical ratings from two sets of raters, with one set of ratings represented in blue and the other in orange. Each dot represents a hypothetical stimulus. Its position represents its average rating in terms of two hypothetical features. In the scatterplots on the left, there is no consistency in ratings across the two sets of raters (blue and orange). In the middle, the consistency in ratings can be captured by one dimension, with ratings orthogonal to this dimension being inconsistent across the two sets of raters. On the right, both dimensions are required to explain the consistency in ratings across the two sets of raters, given that the dots are stable in position along both axes. The dimensions extracted by PCA and factor analytic methods (first row) account only for the distribution of ratings – concatenated or averaged across datasets – and extract the same dimensions regardless of the reliability of judgments of each feature. Thus, PCA is not equipped to identify when an individual category, like fear, is reliably distinguished from every other rated category. By contrast, dimensions extracted by PPCA (second row) are designed to account for the reliability of ratings of each individual feature.

SI References

48. Muto, S. Development of the trait respect-related emotions scale for late adolescence. Shinrigaku Kenkyu **86**, 566–576 (2015).
49. Muto, S., Sugawara, D. & Sugie, M. The conceptual structure of positive emotions in Japanese university and graduate students. Shinrigaku Kenkyu **89**, 479–489 (2018).
50. Nakai, A. Ikari to ikari no kinjigainen no sousatekiteigi no idou oyobi ikari no sousatekiteigi ni eikyo wo ataeta youin [Differences in operational definitions of anger and the approximate concept of anger, and factors affecting the operational definition of anger. Meiji Gakuin Univ. Bull. Psychol. Bull. Psychol. **22**, 13–30 (2012).
51. Higuchi, M., Kuranaga, H., Fukada, H. & Teruya, Y. Mediating mechanisms of embarrassment in non-negative situations. JAPANESE J. Res. Emot. (2012). doi:10.4092/jsre.19.90
52. Kusumi, T. & Komeda, H. Kanjo to gengo [Emotion and language]. in Affective Science 55–84 (Kyoto University Press, 2007).
53. Morishita, A. & Maiya, K. A Study on Japanese Facial Expressions : Analyzing Facial Expressions of Contempt, Disgust and Anger. Tech. Rep. IEICE. HCS **100**, 1–6 (2000).
54. Yamamoto, K. SNOW D18: Japanese Emotional Expression Dictionary [Data set]. http://www.jnlp.org/SNOW/D18. (2018).
